# Wealth, health and inequality in Agta foragers

**DOI:** 10.1093/emph/eoad015

**Published:** 2023-05-15

**Authors:** Abigail E Page, Milagros Ruiz, Mark Dyble, Daniel Major-Smith, Andrea B Migliano, Sarah Myers

**Affiliations:** Department of Population Health, London School of Hygiene and Tropical Medicine, London, UK; School of Health and Social Care, University of Essex, Colchester, UK; Department of Epidemiology and Public Health, University College London, London, UK; UCL Anthropology, University College London, London, UK; Bristol Medical School, University of Bristol, Bristol, UK; Department of Anthropology, University of Zürich, Zürich, Switzerland; UCL Anthropology, University College London, London, UK; BirthRites Lise Meitner Research Group, Max Planck Institute for Evolutionary Anthropology, Leipzig, Germany

**Keywords:** wealth inequality, health, small-scale society, hunter-gatherers, Agta, Philippines, livelihood transition

## Abstract

**Background and objectives:**

There is significant evidence from large-scale, industrial and post-industrial societies that greater income and wealth inequality is negatively associated with both population health and increasing health inequalities. However, whether such relationships are inevitable and should be expected to impact the health of small-scale societies as they become more market-integrated is less clear.

**Methodology:**

Here, using mixed-effect models, we explore the relationship between health, wealth, wealth inequality and health inequalities in a small-scale foraging population from the Philippines, the Agta.

**Results:**

Across 11 camps, we find small to moderate degrees of wealth inequality (maximal Gini Coefficient 0.44) which is highest in the most permanent camps, where individuals engage more heavily in the formal market. However, in both adults (*n* = 161) and children (*n* = 215), we find little evidence that either wealth or wealth inequality associates with ill health, except for one measure of nutritional condition—red blood cell count.

**Conclusions and implications:**

We interpret these results in the light of high levels of cooperation among the Agta which may buffer against the detrimental effects of wealth inequality documented in industrial and post-industrial societies. We observe little intergenerational wealth transmission, highlighting the fluid nature of wealth, and thus wealth inequality, particularly in mobile communities. The deterioration of nutritional status, as indicated by red blood cell counts, requires further investigation before concluding the Agta’s extensive cooperation networks may be beginning to breakdown in the face of increasing inequality.

## INTRODUCTION

Research across the evolutionary sciences has demonstrated that the social determinants of health have a long history in primate evolution [1, 2]. Arguably humans have evolved to seek improved social status producing stress responses which have health implications [3–5]. Within population health research, increasing access to wealth and associated social resources is widely considered to have positive implications for health and wellbeing, particularly in large-scale post-industrial nation states [6, 7]. However, high levels of wealth and income inequality are argued to directly and negatively impact population health [8, 9]. Research has provided correlative evidence that income inequality, a macro-level determinant measuring inequality across individuals within a population, is harmful for longevity [10, 11], stature [9], infant mortality [12] and self-rated health [13], controlling for individual’s wealth. Two parallel phenomena may contribute to this, separating out inequalities in wealth and health. First, populations with higher levels of income inequality may have worse levels of population health compared to more equal populations. A large body of evidence supports income inequality’s detrimental impact on population health in high-income countries [14]. Second, populations with higher-income inequality may also exhibit higher health inequality (e.g. differences in health between persons structured by income, prestige and education [15]). Individuals’ relative rank within a society, directly impacted by inequality, is a leading determinant of health [1]. However, the evidence for increasing income inequality resulting in increasing health inequality is ambiguous [16]. Indeed, the persistence of health inequalities in European countries with advanced welfare states, most notably the Nordic countries, has been described as ‘one of the great disappointments of public health’ [17].

With income inequality either growing or persistently high in most areas of the world [18], it is important to clarify the relations between inequality, health and health inequality; however, associations are complex. For instance, the role of income inequality is likely modified by factors operating in the societal, communal and individual spheres ranging from welfare state systems and social protection policies to population heterogeneity in ethnicity, class and education [19]. As most population health studies have centred on high-income Western societies, it is difficult to disentangle income inequality’s effects from the complex web of macro-level factors driving health and health inequalities. Furthermore, while social status has had a relationship with health throughout human evolution, these relationships may have been altered by globalization, urbanization and systemic issues in high-income contexts [3, 20]. It is informative then to explore whether wealth inequality has similar consequences in a context of less rigid and steep hierarchies [21] and little access to obesogenic diets and reduced physical activities associated with increased market integration [3, 20, 22]. Here, we explore the relationship between wealth inequality (as formalized income is absent) with health and their disparities in the Agta, a small-scale, pre-industrial foraging population in the Philippines.

Evidence for a detrimental relationship between wealth inequality and health and health inequality in small-scale populations is limited. Much of the research has been conducted with the Tsimane, a horticultural-foraging society in Bolivia, producing inconsistent results. For example, a relationship was found between wealth inequality and negative emotions [23], worse nutritional markers [24], higher blood pressure and more respiratory disease [3]. Additionally, inequality in social dominance was associated negatively with several nutritional markers [25]. However, other studies have found no relationship between wealth inequality and nutritional status [3, 19, 26], nor with depression, cortisol, self-rated health or gastrointestinal disease [3]. Further, Undurraga and colleagues [24] reported that inequality was associated with *better* self-reported health and Jäggi, Blackwell and colleagues [3] found a *lower* risk of infectious disease and parasites. Jäggi, Blackwell and colleagues [3] also found that while poorer individuals did not fare worse as inequality increased (i.e. health inequality), wealthier individuals did become *as likely* to suffer respiratory disease as poorer individuals. Ultimately, such work highlights the variability in the relationship between wealth and wealth inequality and health, indicating the need for further exploration in similar small-scale settings. In particular, consideration of the causal pathways linking socioeconomic inequality to health and its inequalities in industrialized populations may improve understanding of the association in pre-industrial contexts.

### Public goods provisioning

A key causal pathway proposed between income inequality and poor health is the societal breakdown of trust [27], social capital, provisioning, norms of reciprocity [9] and civic participation [28]. Higher economic inequality is associated with lower civic integration and participation across Europe [29, 30]. Such relationships may result from inequality increasing heterogeneity, mistrust and fear of free riders, in turn making public provisioning both more difficult and costly [31, 32]. While debates surround the definition and measurement of social cohesion, evidence supports links with health; for example, civically strong counties in the US display lower all-cause mortality rates [33], while across 29 high-income countries social cohesion positively predicts self-rated health after controlling for individual-level characteristics [34]. Societies with greater social cohesion may foster greater social participation, social support and better diffusion of health information, universally improving health [34]. Social cohesion may also mitigate the effects of disadvantaged socioeconomic circumstances on health; reductions in public goods investment directly impacts population health when those in need are no longer provisioned [19]. This can be directly translated to public provisioning in small-scale societies, where individuals use widespread community networks to maximise returns and address resource shortfalls [35, 36]. Such community provisioning consists of mutualist food production and childcare, food sharing and other forms of reciprocity. These mechanisms may protect the health of all in society, but in particular those with the least resources who are unable to absorb shortfalls [37–39].

### Psychosocial stress

A second proposed key causal pathway is the subjective experience of inequality [11, 40], as psychological stress negatively impacts the cardiovascular, immune and endocrine systems. Chronic psychosocial stress is a risk factor for hypertension [41], which in turn predicts increased cardio-vascular disease, chronic kidney disease and neurovascular disease risk [42]. Psychosocial stress is also causally associated with altered immune function [43, 44]. Psychosocial stress triggers an inflammatory immune response [45], elevating levels of glucocorticoids [5], which bind with lymphocytes, reducing their availability and limiting capacity to respond to immunologic challenges [46, 47]. Furthermore, glucocorticoids, are associated with suppressed T lymphocyte and antibody responses [47]; e.g. volunteers with stronger social ties experimentally exposed to rhinoviruses were less likely to present with a cold [48]. Chronic stress breaks down the HPA’s feedback system, rendering glucocorticoid receptors less sensitive to the HPA’s release of anti-inflammatory cortisol, increasing inflammation-related disease risk [45]. Glucocorticoids are also associated with an influx of neutrophils [46], causing a neutrophil heavy neutrophil-lymphocyte ratio (NLR) [46, 49]. Increased NLRs predict increased risk of all-cause mortality and mortality from heart disease, chronic lower respiratory disease, pneumonia, kidney disease and cerebrovascular disease [50]. Higher psychosocial stress in association with higher-income inequality may increase health inequalities where individuals at the bottom of the socioeconomic ladder are disproportionally stressed [11, 40]. On the other hand, everyone’s health may suffer when those at the top or in the middle face increased pressure to maintain their relative position [10].

### Wealth inequality in small-scale populations

Absence of the two causal pathways in small-scale populations may underlie the inconsistent associations between socioeconomic inequality and poor health and health inequalities in the Tsimane. Wealth inequality may not be associated with a breakdown in public goods provisioning to the degree seen in industrialized contexts. Stronger norms of redistribution, reciprocity and denser kinship networks are documented in contemporary small-scale societies, which may dilute the detrimental health impacts of wealth inequality [19]. This is likely to be particularly salient in foraging populations where the role of wealth inequality at the societal level is minimized because access to wealth is often in flux. Mobile, egalitarian foraging populations like the Agta have culturally embedded mechanisms of redistribution, aided by residential mobility and lack of fixed social hierarchies [51–53]. Thus, in these populations societal wealth inequality may bear fewer health risks than in post-industrialized, hierarchical societies because of the context-specific meaning and distribution of material wealth [25].

Here, we build on previous work in the Tsimane by exploring the associations between wealth inequality and multiple health measures in the Agta. Although mobile foragers are renowned for their egalitarianism [54], quantitative exploration of the factors influencing individual life chances has highlighted fluctuating equality and intergenerational wealth transmission [55]. As previously mobile foragers increasingly accumulate wealth and resources, and integrate into local market economies and hierarchical political systems, inequalities in material wealth and political status emerge [56, 57]. The Agta range between a fully mobile (i.e. frequently changing residential camps and minimal market integration) and largely settled (i.e. residing in permanent villages, engaging in cultivation and wage labour and increasingly accumulating resources) lifestyles [58, 59], making them an ideal population in which to explore the association between wealth inequality and health due to expected emergent wealth inequality.

### Predictions

We assess whether the health of individuals associates with household wealth (at the individual level) and wealth inequality (at the camp level). As relevant literature from subsistence populations is currently sparse, we use expectations from industrialized populations to make predictions (rather than the foraging literature) allowing comparison with industrial populations. First, we test the prediction that wealth positively predicts health, employing a range of health indices (P1). Second, we test the prediction that increasing wealth inequality will be negatively associated with health (P2). Our range of health indices, if associated in the expected direction, would be indicative of (i) a breakdown of community provisioning, resulting in elevated markers of malnutrition (body mass index [BMI] and red blood cell count, capturing anaemia) and (ii) an increase in psychosocial stress, implied by differential immunological responses (white blood cell composition) and blood pressure. Next, we add a *post hoc* interaction term to our models to test for a moderating relationship between household wealth and camp inequality when predicting health outcomes, to assess whether inequality is similarly associated with everyone’s health *or* influences those of lower wealth more, thereby increasing health inequalities. Given previous associations between sedentism and health in the Agta [58, 60], we also assess the predication that wealth inequality will positively associate with different measures of sedentism (P3). Finally, in a *post hoc* analysis to shed light on our findings, we explore the relationship between parental with (adult) child wealth to assess wealth transmission (P4).

## Methods

### Data collection

The Agta from Palanan, Philippines, number around 1000 individuals. Their economy is predominately based on fishing, gathering and hunting in the rich coastal waters, rivers and tropical forests of the Northern Sierra Madre Natural Park. Data collection occurred between April–June 2013 and February–October 2014. We conducted camp censuses and household questionnaires to quantify individual’s age, sex, household composition (number of dependents aged 16 and under), household wealth and key camp traits associated with sedentism. For consistency, we conducted the questionnaire with the mother of each household. This research was approved by UCL Ethics Committee (UCL Ethics code 3086/003) and carried out with permission from local government and tribal leaders. Informed consent was obtained from all participants (or for children aged 16 and under permission was received from a parent), after group and individual consultation and explanation of the research objectives in the indigenous language, Paranan.

#### Wealth and wealth inequality

The household questionnaire quantified belongings owned based on a list of the 10 most frequently owned items for which we knew the monetary value (Supplementary Table S1). We asked each household how many they owned of each of these objects (e.g. radios, spears guns, air guns, cooking equipment). The cumulative value was transformed into GBP, giving us a measure of an individual’s ‘*household wealth*’. Wealth was age-corrected by the average age of the parents using a linear model predicting household wealth by age and age squared [3, 21].The age-corrected household wealth variable became the first exposure variable, and also used to create a ‘*mean camp wealth’* variable, allowing us to account for camp-level differences in wealth. The second exposure variable—‘*wealth Gini Coefficient’*—was computed at the aggregate level (per camp) by transforming the age-corrected wealth variable to ensure the base value was a zero using the R package *ineq* [61], with finite sample correction. Possible scores range from 0 to 1, where 1 denotes perfect inequality (i.e. all wealth is held by one household). One camp comprised of a single, extended, household giving this camp a Gini Coefficient of 0; while the inclusion of this camp reflects reality, it does stand apart. We include this camp in all analyses presented below, however, sensitivity analyses are presented in Supplementary Tables S47–83 demonstrating results are unchanged with this camp’s exclusion, except for one model discussed below.

#### Covariates

Camps were divided into either settled (permanent structures, larger camp sizes and presence of infrastructure such as a drinking well or church) or mobile (temporary shelters, smaller camp sizes and frequent residential turnover), making a binary *‘camp settlement’* variable (1 = settled, 0 = mobile). We visited each camp three times over the 2-year period, allowing us to create a ‘*residential mobility’* (1 = mobile, 0 = settled) measure; households were defined as mobile if they had moved camp at least once. We also counted the number of individuals within the camp (‘*camp size’*) and measured in kilometres the distance between each camp and the nearest town, Palanan, creating a *‘distance to market town’* variable. Finally, to capture market integration we computed the mean proportion of activities spent in wage labour by adults, based on camp scan observations documented elsewhere [59].

#### Health markers

We conducted a health survey in collaboration with local health care professionals, including on-the-spot blood composition analysis (using capillary whole blood on HemoCue Hb 301 and HemoCue WBC DIFF systems to capture red blood cell (RBC) count and white blood cell (WBC) differentials), the measurement of blood pressure (diastolic and systolic, DBP and SBP, respectively) and anthropometrics. The WBC differential captured prior or current viral (lymphocytes), bacterial (neutrophil), and parasitic (eosinophil) infection and the NLR [62].

Height and weight were measured for all adults and children in the sample. Height was measured to the nearest millimetre using a Harpenden anthropometer; children unable to stand were measured on the floor. Weight was taken to the nearest 0.1 kg on bathroom scales, used on a hard, flat surface with subjects wearing light clothing and no shoes. For children unable to be weighed by themselves, we first weighed their parent, and then the parent holding the child and subtracted the parental weight. From these measures, we used the World Health Organization (WHO) age- and sex-specific growth standards [63, 64] to compute BMI z-scores using the R package *zscorer* [65].

### Analysis

Our predictions were tested using single level linear regressions (for the camp level analyses) and linear mixed-effect models for the health outcome and wealth transmission models (for the analyses of individuals nested within camps and households). All modelling was conducted in R Version 4.0.3 [66]. using the *glmmTMB* package [67].

#### P1 and P2: Household wealth postively predicts health and increasing wealth inequality is positively associated with ill health

Two mixed effect models were run for each of the nine health metrics, one for adults (aged 16 and above, *n* = 161) and one for children (aged under 16, *n* = 215), with the exception of DBP and SBP which was only measured in adults. Sample sizes varied slightly by health outcomes due to missing observations (Table 1). For many variables, missingness was low—adult BMI 2.2%, child BMI 5.17%, adult RBC 6.18% and child RBC 5.6%—which is unlikely to bias responses. However, missing was higher for the white blood cell counts (adults 20.23%, child 12.07%) and blood pressure measurements (22.47%). Sample descriptives in Supplementary Table S2 and S3 indicate the distributions for household and camp characteristics do not diverge from the non-missing sample, limiting bias concerns. One camp (of 17 adults and children) was removed from the analysis because no wealth data collected due to oversight in data collection. The models accounted for the hierarchically nested nature of the sample (individuals within households residing in camps): adult models—72 households within 11 camps; child models—65 households within 11 camps. To increase variance and minimise standard errors, we ran the RBC and WBC models with the exposure modelled continuously; logistic models based on the binary outcomes can be found in the Supplementary Result tables.

**Table 1.** Descriptives for the continuous variables for adults (*n* = 178) and children (*n* = 232)

	Mean	SD	Min	Max	Missingness
	Adults				
Age	37.51	4.47	16.00	80.00	0
BMI	18.57	2.04	11.93	23.81	5
DBP	80.00	7.76	60.00	90.00	44
SBP	100.00	8.64	80.00	120.00	44
Neutrophil count	4.70	1.43	0.90	10.90	40
Lymphocyte count	2.93	0.99	1.20	6.90	40
Eosinophil count	1.03	0.78	0.00	5.10	40
Red blood cell count	123.20	25.86	32.00	170.00	15
NLR	1.49	0.68	0.47	4.19	40
Household wealth	73.15	29.66656	0	122.9	24
	Children				
Age	6.99	4.47	0.00	15.50	0
BMI-z	-0.73	1.58	−3.62	9.39	12
NLR	1.06	0.62	0.30	5.20	30
Neutrophil count	4.48	1.91	1.60	15.50	30
Lymphocyte count	4.74	1.65	1.50	10.70	30
Eosinophil count	1.68	1.19	0.00	7.20	30
Red blood cell count	114.50	18.31	56.00	153.00	14
	Camp level				
Gini Coefficient	0.23	0.12	0.00	0.44	0
Mean camp wealth	53.84	14.74	37.85	87.87	0
Distance to town	18.98	5.43	7.98	26.00	0
Wage labour	0.23	0.17	0.00	0.47	0
Camp size	36.27	22.97	12.00	77.00	0
Mean camp relatedness	0.14	0.07	0.07	0.30	0

BMI = body mass index; DBP = diastolic blood pressure; NLR = neutrophil-lymphocyte ratio; SBP = systolic blood pressure. Units for neutrophil, lymphocyte and eosinophils is 10^9^/L, for RBC is g/L

Household wealth and camp Gini coefficient were set as exposures. To explore if increasing wealth inequality resulted in increasing health inequality, the models were then re-run including an interaction term between camp level Gini coefficient and household wealth. Several models failed to converge with both camp and household structures as random effects, given the limited number of individuals within some households; in this case the household level was removed.

#### P3: Wealth inequality is positively associated with sedentism

We ran six linear regression models to assess if different aspects of sedentism positively predicted the camp (*n* = 11) wealth Gini coefficient. The exposure variables were the following: (i) camp settlement, (ii) distance to market town (km), (iii) mean camp wealth, (iv) mean camp proportion of activities spent in wage labour, (v) camp size and (vi) mean camp relatedness. Camp settlement, engagement in wage labour, camp size and household wealth increase with sedentism, while distance to market town decreases (Supplementary Fig. S1).

#### P4: Parental wealth positively predicts the wealth of adult children

Finally, two linear mixed effect models (with camp as a random effect) were run to assess if independently residing adult children’s household wealth was predicted by the wealth of the parents of either (i) the woman or (ii) the man of the household. We split the adult sample by sex so each household was represented only once in each of the women’s (*n* = 23, *n*_camps_ = 8) and men’s (*n* = 16, *n*_camps_ = 7) samples. The reduced sample is a result of us not having collected data on the parental wealth of each adult. For this analysis, two additional variables were created: the outcome variable ‘*parental household wealth’* and the potential control term ‘*parental mean camp wealth’* to capture effects stemming from parents being located in relatively wealthier camps or not.

#### Model selection

We use directed acyclic graphs (DAGs, Supplementary Figs S2–S4), constructed using *dagitty* [68] and tested using the *LAVAAN* package [68] to select our models (see Supplementary Information for more details). The following variables, in addition to our outcome measures, are included in our DAGs: camp settlement, camp size, mean camp wealth, wealth Gini Coefficient, camp mean proportion of activities in wage labour, distance to market town, individual’s sex and age, residential mobility, household wealth and number of household dependents (aged 16 and below). We then selected the smallest minimally sufficient adjustment sets to adjust for in our models [69]. For the health models the control variables retained were camp settlement and residential mobility. The control terms included in the camp models varied by exposure: camp settlement controlled for distance of market town and mean camp wealth, mean camp wealth and proportion of time in wage labour controlled for distance to market town, camp size controlled for camp settlement and camp settlement and distance to market town contain no additional control variables. The wealth transmission models included no additional control variables.

The following results focus on our exposure variables, full model results are provided in the Supplementary Information alongside further ethnographic and methodological information. The full dataset and code to replicate these analyses can be found at https://osf.io/hqzm8/.

## RESULTS

### Descriptives

In the child sample (aged 0–16 years), there were 40.95% girls (*n*_Girl_ = 95, *n*_Boy_ = 137), reflecting the sex bias in the under 16s [70]. In the adult sample (aged 16–80 years), there were 48% women (*n*_Women_ = 85, *n*_Men_ = 93). Camp wealth Gini coefficients ranged from 0 to 0.44, with a mean of 0.23 (SD = 0.12), placing the Agta in line with other foraging groups worldwide [55], and only slightly lower than reported for horticultural-foragers [19, 56]. Adults tended to be underweight (BMI < 18.5 [71]), with a mean BMI of 18.57 (SD = 2.04, *n* = 173); 37.6% were ‘slightly underweight’ (BMI 16–18.49) and 8.67% ‘severely underweight’ (BMI < 16). Compared to the WHO reference population, the average BMI z-score for children was −0.732 (*n* = 220), falling below the reference median; by WHO definitions, 12.27% were ‘thin’ and 4.10% were ‘severely thin’ [72]. The average adult blood pressure (*n* = 93) was 100/80 (falling into the ideal range); all individuals had normal SBP (SBP 80–120), while 23.12% had DBP indicative of high blood pressure (DBP 90 and above) (Table 1).

#### P1: Does wealth positively predict health?

We find no compelling evidence of a positive relationship between household wealth and any adult or child health outcomes (Table 2). The point estimates highlight small effects and are not consistently in the predicted direction; DBP and eosinophil white blood cell counts were slightly raised in wealthier households in adults, while RBC was lower in children from wealthier households, for example. All 95% confidence intervals spanned 0.

**Table 2.** Results from mixed-effect health outcome models for adults and children. Sample size varies by outcome variable, please see methods. Models contain random effects for camp and household.

	Camp wealth Gini				Household wealth			
	β	*P*	2.5% CI	97.5% CI	β	*P*	2.5% CI	97.5% CI
Adults (aged 16 + years)								
BMI	−0.569	0.751	−4.083	2.945	0.002	0.635	−0.008	0.013
DBP	−10.630	0.188	−26.443	5.183	0.023	0.327	−0.023	0.069
SBP	−9.862	0.286	−27.970	8.247	−0.007	0.796	−0.060	0.046
Neutrophil	−0.642	0.682	−3.712	2.428	0.003	0.559	−0.006	0.011
Lymphocyte	0.606	0.558	−1.423	2.636	0.000	0.882	−0.007	0.006
Eosinophil	−0.726	0.352	−2.255	0.803	0.003	0.175	−0.001	0.007
NLR	−0.632	0.400	−2.104	0.840	0.001	0.747	−0.003	0.005
RBC	−58.836	0.015	−106.450	−11.222	0.003	0.970	−0.135	0.141
Children (aged less than 16 years)								
BMI (z-score)	0.985	0.405	−1.335	3.306	0.003	0.424	−0.004	0.010
Neutrophil	0.669	0.717	−2.948	4.287	0.002	0.732	−0.009	0.013
Lymphocyte	−3.007	0.435	−10.550	4.536	−0.022	0.208	−0.056	0.012
Eosinophil	0.173	0.877	−2.011	2.356	0.003	0.371	−0.004	0.010
NLR	−0.420	0.513	−1.679	0.839	0.000	0.832	−0.004	0.004
RBC	−38.266	0.037	−74.186	−2.347	−0.053	0.361	−0.168	0.061

BMI = body mass index; DBP = diastolic blood pressure; NLR = neutrophil-lymphocyte ratio; RBC = red blood cells; SBP = systolic blood pressure. Units for neutrophil, lymphocyte and eosinophils is 10^9^/L, for RBC is g/L. Models controlled for camp settlement and residential mobility. Full results can be found in the Supplementary Information.

#### P2: Is wealth inequality negatively associated with health?

**Figure 1.**
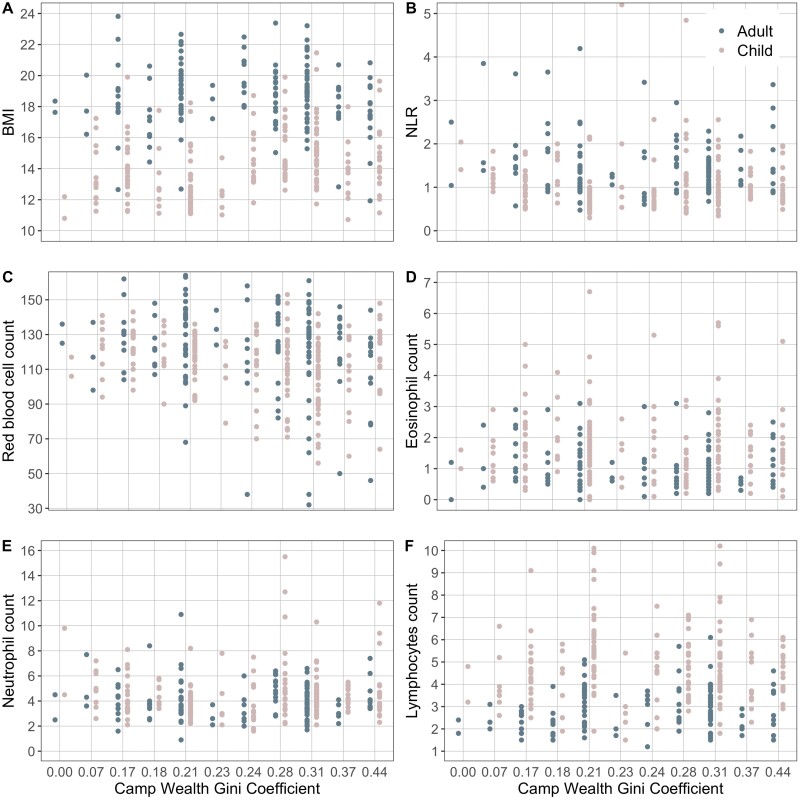
Adult (blue) and child (pink) health metrics—(A) body mass index (BMI), (B) neutrophil-lymphocyte ratio (NLR), (C) red blood cell count (RBC), (D) Eosinophil count, € Neutrophil count and (F) lymphocyte count by camp wealth Gini coefficient. Sample size varies by model, please see methods.

**Figure 3.**
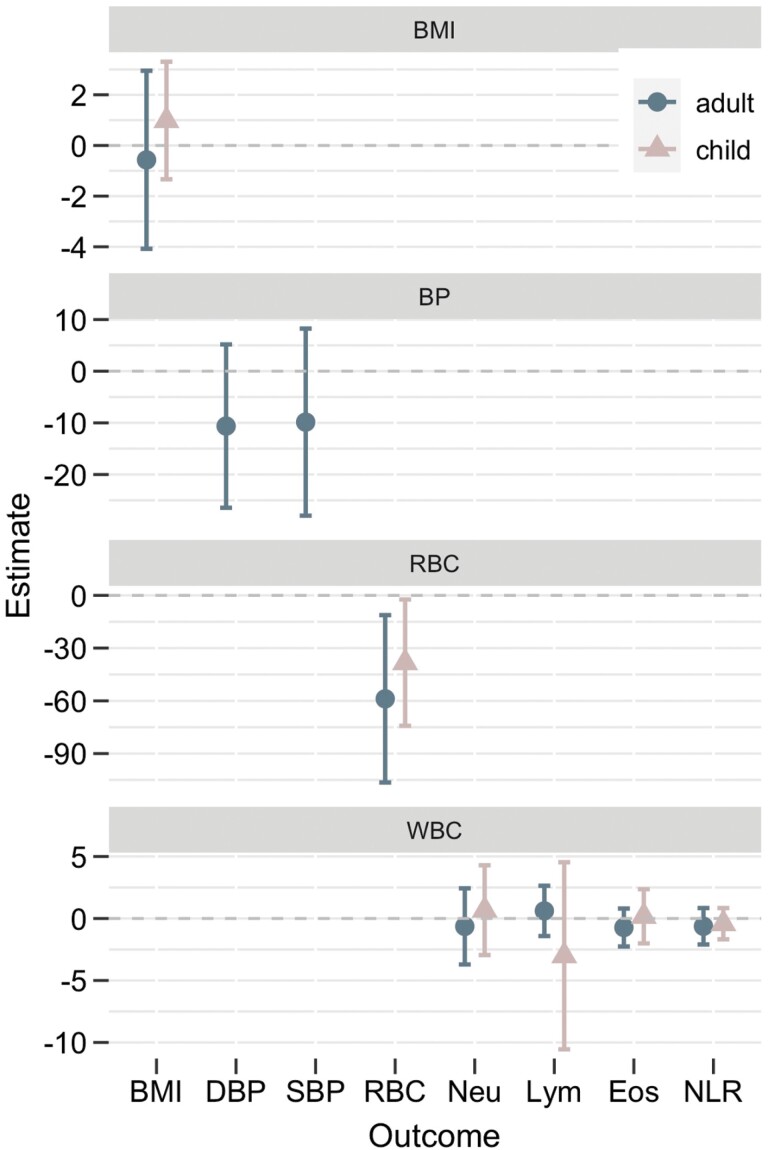
Point estimates for the adult (blue circles) and child (pink triangles) for each of the health outcomes by wealth Gini Coefficient. The dash grey line represents no effect.

We found no compelling evidence of a relationship between wealth inequality and markers of ill health for adults or children, except for RBC (Table 2, Fig. 3). A one-point increase in Gini Coefficient is associated with a 58.84 (95% CI [−106.45, −11.22]) point decrease in RBC in adults and a decrease of 38.27 (95% CI [−74.19, −2.35]) in children. Given haemoglobin values below 130 g/L for men, 120 g/L for women and 110 g/L children are considered indicative of anaemia, these results reflect a biologically significant result. Camps with Gini Coefficients of 0.2 and above have a large number of individuals with low and extremely low levels of haemoglobin (Fig. 1C).

**Figure 2.**
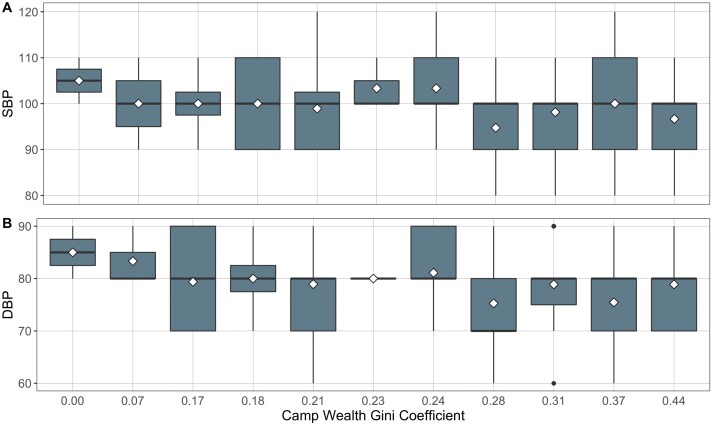
Relationship between adult blood pressure and camp wealth Gini Coefficient divided into (A) SBP and (B) DBP (*n* = 118). Diamonds are means, lines medians.

For all other models, no such strong trends were apparent. The point estimates were often in the opposite direction to that predicted: those for DBP, SBP, neutrophil and eosinophil counts and NLR are negative in the adult models, and BMI, lymphocyte count, and NLR negative in the child models. Furthermore, other than for RBC, all 95% confidence intervals spanned 0 (Fig 1 and 2).

Interaction models indicate no compelling evidence for an association between health inequality and wealth inequality. Across all bar two models, the interactions between Gini coefficient and household wealth were statistically non-significant, and the direction of the effect was inconsistent (Supplementary Tables S4–39). In the DBP model, DBP was higher in individuals from wealthier households in the most equal camps (interaction beta = −0.542, 95% CI [−1.055, −0.029]). In children, eosinophils were highest in the wealthiest children in the most unequal camps (interaction beta = 0.085, 95% CI [0.015, 0.156]). Neither of these trends are in line with predictions, suggesting that the mechanisms involved are unrelated to wealth inequality.

#### P3: Is wealth inequality positively associated with sedentism?

**Figure 4.**
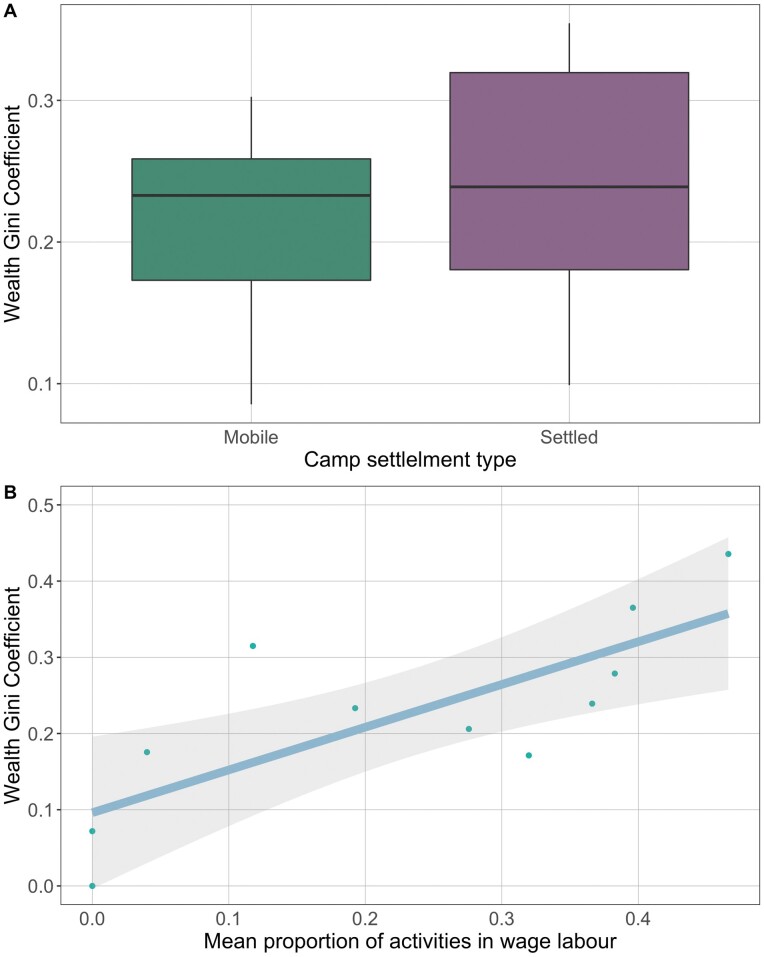
Relationship between camp wealth Gini Coefficient and (A) camp settlement and (B) mean proportion of activities in wage labour, with 95% confidence intervals (*n* = 11).

Camp permanence (i.e. mobile vs settled) positively predicted wealth Gini coefficients (β = 0.164, *P* = 0.056, 95% CI [−0.004, 0.331]), as predicted (Fig. 4). However, this result was conditional on the inclusion of the camp consisting of one household creating a Gini Coefficient of 0. When this camp is removed from the analysis (reducing power, *n* = 10) this relationship disappears (Supplementary Table S76). There is strong evidence, however, that camps with the highest proportion of activities spent in wage labour had statistically higher Gini Coefficients, (β = 0.540, *P* < 0.001, 95% CI [0.249, 0.832]; Fig. 4B). Other measures had inconsistently directed point estimates and either very small effects, and 95% CIs spanning or hitting 0, or a larger effect size but CIs heavily overlapping 0.

#### P4: Does parental wealth positively predict the wealth of adult children?

For both women and men, the effects are small, inconsistent and the CIs very wide: women – β = −0.115, *P* = 0.529, 95% CI [−0.471, 0.242]; men – β = 0.165, *P* = 0.687, 95% CI [−0.639, 0.969].

## DISCUSSION

In the past, mobile foraging populations are often presented as egalitarian, lacking wealth inequality. Indeed, social norms expected to limit the development of inequality have been documented, with groups demonstrating ‘levelling’ behaviours, e.g. talking down ‘big shots’ and demand sharing [52]. However, contemporary assessments document the presence of wealth inequality among foragers worldwide [55]; here, we find the Agta to be in line with other foragers, with wealth Gini Coefficients ranging up to 0.44, with a mean of 0.23. These estimates overlap the income Gini Coefficients of the most equal large-scale industrial and post-industrial societies, ranging between 0.25 and 0.29 [14], though are less than those for wealth: Netherlands at 0.64, Slovakia 0.68 and Denmark 0.71 [73]. Nevertheless, given the Agta’s upper range, the *a priori* assumption that wealth inequality would predict health disparities seems reasonable. However, across a range of health outcomes, we find only one strong relationship—that wealth inequality negatively predicted red blood cell count. We find no other evidence that wealth inequality impacted child or adult health. Likewise, there is little evidence that health inequality was increased in the most unequal camps. Of course, as with many anthropological studies, we are limited by the sample size and our null results may be due to low power. We cannot argue we have evidence *against* the hypothesis that rising wealth inequality results in deteriorating health, yet many point estimates tended to go in the opposite direction than predicted, creating no consistent trend in support of the hypothesis, regardless of study power. To help understand these diverse results, it is useful to consider the pathways which underpin their relationship with wealth inequality.

### Psychosocial stress

We expected to find some signal of a relationship between wealth inequality and measures of ill health which capture psychosocial stress, since there is strong and consistent evidence that blood pressure is highest in the most unequal Tsimane communities [3]. The Agta do not lack elevated blood pressure (of which heart disease or hypertension are major consequences [74]). Indeed, raised blood pressure has been consistently reported in small-scale populations undergoing livelihood transitions [41, 75]. However, we find no strong evidence for a relationship between blood pressure and wealth inequality, a pattern mirrored by NLR. In regard to these proxies then, our results suggest that inequality at the community level was not a source of psychosocial stress. Given the importance of socioeconomic circumstances [3, 40, 76], and negative behavioural and stress responses [2, 4], for human fitness these results are perhaps surprising and deserve further exploration with detailed psychosocial measures.

Given the generally null results and heterogeneous point estimates, we *post hoc* investigated generational wealth permanence in the Agta. Borgerhoff Mulder and colleagues [21] argue it is where wealth has large fitness consequences that wealth is passed from parents to children and inequality accumulates, as seen in pastoralist and agricultural societies with ‘strong’ inheritance norms. In contrast, in relatively egalitarian and mobile populations like the Agta, norms of redistribution may ensure both individual’s wealth and camp-level inequality are transient. Consequently, individuals in more unequal communities at a given time do not suffer long-term ill effects of inequality. Supporting this, we find no evidence of an association between wealth across generations, suggesting that wealth may not transmit from parents to adult children (though there are other processes at play which can result in a correlation between parental and child wealth [21, 55]). Household wealth may lack importance, or social meaning, within the Agta due to its fluid nature, thus lacking wealth and residing in more unequal camps may not be physiologically or psychologically stressful.

The mobile nature of foraging populations like the Agta is also likely an important mechanism facilitating these relationships. Since mobile populations are able to ‘vote with their feet’ [52], individuals can walk away from camps where they have poor status and join more ‘status similar’ camps. Thus, with the lack of vertical transmission of wealth and ability to change their social surroundings, the permanence of exposure to wealth inequality is likely significantly reduced. Comparatively, in large-scale societies social mobility is increasingly limited [77, 78], leading to concentrations of wealth in groups who benefit from monopolising resources over other groups. Such persistent inequalities have greater stress potential. This of course, raises a question about whether the inequality we measure now captures an individual’s life course exposure to inequality [79]. Only longitudinal work can untangle these pathways, however, arguably the current fluid nature of Agta camps likely dampens any impact of inequality.

### Public goods provisioning

The Agta, as largely (but not exclusively) fisher-foragers, deal with the necessary stochasticity in daily foraging returns (as well as household shortfalls in caring) by creating extensive networks of cooperation with family and other camp members [51, 80]. These systems of reciprocity act as social insurance to buffer individuals from risk, allowing individuals to meet their energetic needs, levelling out any natural shortfalls [35, 36]. Increasing wealth, and in particular increasing wealth differentiation within the community, can break down these relationships (see [51] for discussion) meaning individuals are exposed to shortfalls. This trend may underpin the finding that individuals in the most unequal camps are at increased risk of iron-deficient anaemia, as measured in haemoglobin levels (i.e. RBC). Haemoglobin levels reflect micro-nutritional quality, capturing variability in the type and quality of an individual’s diet, which is readily impacted by social provisioning. Similar results in terms of individuals nutritional status (measured by BMI, muscle mass and skin fold thickness) and wealth inequality have been found in prior studies [3, 24], but not consistently [3, 19]. However, our second measure of nutritional status, BMI, showed no such relationship with inequality, complicating this interpretation. Likewise, though poor nutritional status predicts other instances of ill health, our white blood cell analysis also finds no links with inequality. Additionally, under the proposed pathway it is reasonable to expect those with the *least* wealth in the most unequal camps to suffer most (as they have the least wealth to buffer themselves and cooperation is least available to them), yet we see no such relationship. What may underpin this complex picture is the lack of evidence that settlement negatively impacts traditional cooperative networks in the Agta.

Rising wealth inequality does not necessarily entail a breakdown of cooperation in the small-scale subsistence populations. For instance, Agta mothers in settled camps receive the same or more childcare compared to mobile camps [81]. Furthermore, reciprocal sharing in an experimental game was higher in settled camps, likely aided by the increased likelihood of future interactions between dyads [82]. Likewise, in the Tsimane, gift giving, help with labour and intensity and breadth of food sharing has been found to *increase* as village inequality increases [19, 83, 84]. Experimental resource allocation games with the Agta find sharing is often based on recipient need [85], as is cooperation in childcare [86], and households are more likely to receive food from the wider camp on days they do not produce anything themselves or receive anything from their most frequent exchange partners [87], supporting a needs-based redistribution of resources that protects health. Ultimately, the robustness of these traditional sharing networks in the face of market integration, as documented elsewhere [84], may explain our overwhelming null results. However, our previously work has only explored the relationship between cooperation and settlement, *not inequality*. Settled status and Gini Coefficient have only a weak association, and comparatively market integration (i.e. engagement in wage labour) is a stronger predictor of wealth inequality and bears exploration in relation to cooperation. Future work should also directly explore the relationship between wealth inequality and cooperation; such work may shed light on our haemoglobin results.

### Health, wealth and its inheritance

Household wealth has little influence over health outcomes. This is in opposition to findings in the Tsimane [3], where household wealth was generally associated with beneficial health outcomes. However, previous Tsimane studies point to a lack of consistency in results, with self-reported health lower in wealthier communities [88] and increased income from market sources associated with higher cortisol levels [89]. Furthermore, a recent study in the Turkana (pastoralists from north-western Kenya) found that while absolute material wealth was positively correlated with improved self-reported health in those living rurally, this relationship was reversed (in terms of cardiometabolic markers of health) with urbanization and greater market integration [20]. Ultimately, the relationship between wealth and health is appears to be context specific.

### Limitations

The key limitation of this study is the small sample, which unavoidably increases our Type II error rate. This issue is common in studies of small-scale societies, as the populations the sample originate from are often small (e.g. the Agta from Palanan number around 1000 individuals) and intensive data collection in remote locations is time consuming and resource expensive. The degree of concern should be dependent on expected effect sizes, and while previous analyses using the same data set have demonstrated clear relationships between livelihood transitions and health [58, 60], work in the Tsamine point towards small to moderate effect sizes associated with wealth and wealth inequality [3]. Nonetheless, future work should be conducted in larger samples, across a range of other subsistence-level populations to examine the degree to which our results replicate. Another limitation is the accuracy with which pathways between wealth inequality and health and health inequality are proxied by our markers of health. To interpret our results, we have rested heavily on the assumption that cooperation networks remain strong in the Agta, redistributing wealth and resources among the population; however, this requires quantification, ideally with longitudinal data. Finally, in keeping with the literature from larger societies, here we have only explored one dimension of wealth (material). This ignores foragers reliance on social network wealth, which may be a more culturally relevant measure of wealth among the Agta.

## CONCLUSION

We find little evidence that wealth inequality negatively impacts multiple and varied health outcomes in Agta adults or children. Facets of Agta society appear likely candidates to buffer against the detrimental effects of wealth inequality documented in industrial and post-industrial societies. An apparent lack of intergenerational wealth transmission points to the fluid nature of unequal experience. While an increase in iron-based anaemia in the most unequal camps is potentially suggestive of a loss of social provisioning and reciprocity, most other evidence points to needs-based sharing effectively protecting against ill health. This indicates that the social norms of redistribution are currently robust to emerging wealth inequality. Overall, our results support the contention that wealth inequality, at least in association with the earliest stages of transition in a mobile egalitarian foraging community, does not *necessarily* lead to adverse health outcomes.

Much insight can be gained by examining highly cooperative small-scale societies, since they allow us to examine the mechanisms that mitigate the costs of rapidly increasing income inequality. These results highlight the potential for societal behaviours and public policies to disrupt the causal pathways linking wealth inequality with health and health disparities. Among European countries, those with the most generous welfare states and greatest levels of public provisioning, not only have lower levels of inequality, but tend to also demonstrate better population health. Nonetheless, health inequality often persists, something apparently absent in the Agta. Our results add weight to the conclusion that welfare regime types and stages of social and economic development can moderate the relationship between wealth inequality, health and health inequality. These findings are likely to be particularly salient to populations currently transitioning from small-scale subsistence economies, into Westernized market-based economies, pointing to the importance of maintaining cooperative networks in communities as they become increasingly market-integrated.

## Supplementary Material

eoad015_suppl_Supplementary_Material
