# Supplementary material for: Wealth, health and inequality in Agta foragers
Source: Evol Med Public Health. 2023 May 15;11(1):149–62. doi: 10.1093/emph/eoad015 (PMC10237286; doi:10.1093/emph/eoad015)
Supplement: eoad015_suppl_Supplementary_Material [file eoad015_suppl_supplementary_material.docx]

**SI: Wealth, health and inequality in Agta foragers**

**Authors**

Page, A. E.^1^, Ruiz, M.^2,3^, Dyble, M.^4^, Major-Smith, D.^5^, Migliano, A. B.^6^ and Myers, S.^4,7^

**Affiliations**

^1^ Department of Population Health, London School of Hygiene and Tropical Medicine, UK

^2^ School of Health and Social Care, University of Essex, UK

^3^ Department of Epidemiology and Public Health, University College London, UK

^4^ UCL Anthropology, University College London, UK

^5^ Bristol Medical School, University of Bristol, UK

^6^ Department of Anthropology, University of Zürich, Switzerland

^7^ BirthRites Lise Meitner Research Group, Max Planck Institute for Evolutionary Anthropology, Leipzig, Germany

**Ethnographic information**

Our study focuses on a population of approximately 1000 individuals who reside within the municipality of Palanan, Isabela Province. Living in small settlements (of 30–100 individuals) at the coast and along rivers, the Palanan Agta are mainly fisher-foragers, with variable emphasis on hunting, combined with cultivation and wage labour. The resources on which they subsist are situated in the Northern Sierra Madre Natural Park (NSMNP), the Philippines' largest protected area (Minter, 2010) that consists of a mountainous tropical rainforest and includes the coastal beaches, coral reefs and the marine eco-system of the Pacific Ocean. Having indigenous status, the Agta hold user and settlement rights in the park.

Similar to many immediate-return hunter-gatherer societies worldwide the Agta follow a bilateral descent and residence system, which maintains a large and flexible kinship network (Dyble et al., 2015; Griffin, 2012; Minter, 2010; Peterson, 1978). Having such a large kinship base allows easy access to collectively held land as family groups are mobile, and often move between different camps on a regular basis. On average, households move once every 10 days, but this varies according to degree of sedentarization. Some households moved regularly between nearby sites (a trend noted by Peterson (1978)), while some never moved. When this data is transformed into a binary variable of either moved once or never moved, we found that 27.5% out 444 households were witnessed to move camp at least once. Accordingly, we find that 26.8% of 444 households reside in mobile camps, comprised of temporary housing (lean-tos) without the presence of infrastructure such as water pumps or the presence of the church.

The Agta have long-standing interactions with their neighbors, a common feature of many Agta and Aeta groups in the Philippines. Peterson (1978) argues it was the disruption caused by the Japanese occupation that caused non-Agta farmers to spread out into the municipality. However, as there is no road or easy route to Palanan, external pressures (extractive industries, land grabbing and clearing, resource destruction and migration) have been significantly reduced for the Palanan Agta compared to populations to the south of the NSMNP (Griffin, 2012; Minter, 2010). Extractive technologies, such as mining and logging were during the data collection period, absent from Palanan, in complete opposition to Dinapigue and Dilasag to the south.

To measure the amount of time different individuals spent in foraging and non-foraging activities we conducted daily camp scans to record activity patterns based on spot observation techniques [9,10]. We categorised everyone's activity (broadly separated into foraging, non-foraging, domestic tasks, childcare and leisure time) at the allocated time, and if they were out of camp their reported activity was recorded. To produce an unbiased time sample, the first scan time was rotated daily and then three more scans were conducted every four hours from this starting point. From this data, we extracted how long individuals spent foraging (i.e., hunting, fishing and gathering) compared to cash labour and agricultural food production.

The Agta are predominantly marine and river fisher-gatherers, as out of 2,168 work activities 53.6% were spent fishing, 2.9% hunting, and 20% gathering. Non-foraging activities consist of cash labour (9.5% of activities), cultivation (12%) and trade (2.1%). Thus, the Agta continue to rely heavily on foraging modes of subsistence (76.5%) versus non-foraging activities (23.5%). Nonetheless, this varies significantly according to gender, location (coastal or inland ecologies) and degree of sedentarisation. For instance, mobile groups spent only 3.5% of their work activities in cultivation and 0% of activities in cash labour and trade. Comparatively, settled groups spend 11.6%, 10% and 2.5% of their work activities in cultivation, cash labour and trade, respectively. As a result, while settled groups spent only 69.3% + 36.1% of time in ‘foraging’ activities (the combination of gathering, fishing and hunting), mobile groups spent a mean of 96.2% + 10.3%. Figure S1 shows significant correlations between proportion of food produced from foraging (gathering, fishing and hunting) and key ‘transition’ traits.

**Figure S1:** Correlation plot for six key ‘transition’ variables and wealth Gini coefficient. The stronger the shade, the stronger the correlation, represented on the bar legend. The figure in the centre of each square is the p-value associated with the correlation. Variables: R = mean camp relatedness; Wealth = mean camp wealth; Distance = distance to market town; Gini = wealth Gini coefficient; Labour = mean camp proportion of activities spent in wage labour; Settled = camp settlement; Size = camp size.

**Extended data collection**

*Household wealth*

To create an emic based list, we first sought to establish the most important items from a sub-sample (*n* = 16) of households. We asked each household to name 10 of the most important belongings an Agta could own. Based on this we created a list of 14 household items that were mentioned the most frequently. This list was then shown to each household, asking whether they had these items and if they did, how many did they have. From this list we estimated the monetary value of each object in Philippine Peso, which was converted to GBP. Two items (hunting bow and googles) were removed during this process as they were constructed by the Agta from locally available resources, making it impossible to attach a monetary value to them. The object, count and monetary value can be found in Table S1.

**Table S1**: List of household objects and their weighting used in creation of household belonging variable.

| **Item** | ***n*** | **Peso** | **GBP** |
| --- | --- | --- | --- |
| Blanket | 37 | 350 | 5.395406 |
| Cups | 65 | 25 | 0.385386 |
| Air gun | 5 | 3000 | 46.24634 |
| Kettle | 45 | 450 | 6.936951 |
| Knife | 65 | 120 | 1.849854 |
| Mat | 15 | 450 | 6.936951 |
| Net | 12 | 450 | 6.936951 |
| Plates | 93 | 45 | 0.693695 |
| Cooking pot | 123 | 350 | 5.395406 |
| Radio | 4 | 450 | 6.936951 |
| Speargun | 35 | 110 | 1.695699 |
| Spoon | 50 | 20 | 0.308309 |

The ‘spear-guns’ that the Agta use are not mass-produced items but are home constructed comprising of a metal arrow and piece of rubber to catapult the spear across a short distance into a fish. They are widely owned as cheaply made with readily available materials.

*Blood Collection Protocol*

The standard protocol for blood collection is as follows. A blood sample, obtained by a Haemolance Normal Flow lancet, of approximately 10 μL is drawn into the cavity of the specially designed microcuvette by capillary action.  Following best procedure, blood was always taken from the end of the middle or ring finger on the right hand (Morris et al., 1999). The first two or three drops were wiped away. Clotted samples were discarded, and the sample was always taken within one minute prior to analysis.  If multiple samples were required (due to a lost sample, clotting or lack of flow) a different finger was used each time as skin puncture causes the body’s defense system to increase the number of WBC close to the wound, affecting the overall measurement.  The blood flow was never encouraged by squeezing due to the altering effect this has on the blood sample (Morris et al., 1999).  The microcuvette was then placed into the analyzer.

The WBC analysis was conducted on HemoCue^©^ WBC DIFF for in-vitro of white blood cell composition in capillary blood.  The WBC DIFF produces a full white blood cell differential within five minutes using staining and image analysis within the analyzer.  As a portable, battery operated system WBC DIFF provides immediate values for total white blood cell count and a differential count of the five main leukocytes (neutrophil, lymphocyte, monocyte, eosinophil and basophil) each of which have specific functions and morphologic appearance, making classification possible (Greer et al., 2013). All quality control studies of the WBC DIFF system find the results to be confidentially repeatable with small standard deviations between tests and produce similar results to other methods (Bentley et al., 1993; Greer et al., 2013; van Assendelft, 2002). Traditionally the collection of biological samples has been very difficult from foraging groups due to their remote locations and the difficulties of storing biological samples in often hot conditions. The HemoCue systems successfully overcome these limitations by allowing the blood differential to be made immediately and then the sample discarded.

**Missing data**

To explore whether the slight variation in sample size between modelled outcomes may have biased our results, we have compared the descriptive characteristics of individuals with missing data for each of the outcomes. The results are provided in Table S2 for adults and S3 for children.

**Table S2:** Settlement characteristics for adults with missing health outcome data. P-values are produced from a comparison of means between the different groups.

|  | **BP NA**  **(N=46)** | **Not missing (N=94)** | **RBC NA**  **(N=3)** | **WBC NA (N=24)** | **Total**  **(N=167)** | **p value** |
| --- | --- | --- | --- | --- | --- | --- |
| **Camp settlement** | | | | | | |
| **Mobile** | 10 (21.7%) | 26 (27.7%) | 0 (0.0%) | 10 (41.7%) | 46 (27.5%) | 0.229 |
| **Settled** | 36 (78.3%) | 68 (72.3%) | 3 (100.0%) | 14 (58.3%) | 121 (72.5%) |  |
| **Distance to market town** | | | | | | |
| **Mean (SD)** | 16.64 (5.34) | 18.76 (4.30) | 17.83 (0.58) | 18.15 (5.65) | 18.07 (4.83) | 0.113 |
| **Median (Min, Max)** | 18.50 (2.66, 24.70) | 18.50 (7.98, 26.00) | 17.50 (17.50, 18.50) | 21.20 (7.98, 24.70) | 18.50 (2.66, 26.00) |  |
| **Camp size** | | | | | | |
| **Mean (SD)** | 57.59 (20.49) | 52.47 (23.45) | 74.33 (2.31) | 46.83 (17.54) | 53.46 (22.00) | 0.081 |
| **Median (Min, Max)** | 73.00 (21.00, 77.00) | 54.00 (12.00, 77.00) | 73.00 (73.00, 77.00) | 39.00 (26.00, 77.00) | 54.00 (12.00, 77.00) |  |
| **Mean camp wealth** | | | | | | |
| **Mean (SD)** | 1.71 (0.56) | 1.89 (0.69) | 2.21 (0.75) | 1.82 (0.60) | 1.83 (0.65) | 0.248 |
| **Median (Min, Max)** | 1.66 (0.68, 2.64) | 1.68 (0.68, 3.55) | 2.64 (1.34, 2.64) | 1.68 (0.68, 2.64) | 1.68 (0.68, 3.55) |  |
| **Household wealth** | | | | | | |
| **Mean (SD)** | 1.73 (1.07) | 2.02 (1.06) | 1.99 (0.94) | 1.62 (0.79) | 1.89 (1.03) | 0.248 |
| **Median (Min, Max)** | 1.66 (0.21, 4.80) | 2.05 (0.21, 5.50) | 2.05 (1.03, 2.90) | 1.60 (0.18, 2.90) | 1.82 (0.18, 5.50) |  |
| **Camp wealth Gini** | | | | | | |
| **Mean (SD)** | 0.26 (0.12) | 0.21 (0.12) | 0.20 (0.17) | 0.20 (0.09) | 0.23 (0.12) | 0.099 |
| **Median (Min, Max)** | 0.28 (0.10, 0.40) | 0.18 (0.00, 0.40) | 0.10 (0.10, 0.40) | 0.17 (0.10, 0.40) | 0.18 (0.00, 0.40) |  |

**Table S3:** Settlement characteristics for children with missing health outcome data. P-values are produced from a comparison of means between the different groups.

|  | **BMI NA (N=3)** | **Not missing (N=182)** | **RBC NA (N=3)** | **WBC NA (N=27)** | **Total (N=215)** | **p value** |
| --- | --- | --- | --- | --- | --- | --- |
| **Camp settlement** | | | | | | |
| **Mobile** | 3 (100.0%) | 48 (26.4%) | 2 (66.7%) | 8 (29.6%) | 61 (28.4%) | 0.018 |
| **Settled** | 0 (0.0%) | 134 (73.6%) | 1 (33.3%) | 19 (70.4%) | 154 (71.6%) |  |
| **Distance to market town** | | | | | | |
| **Mean (SD)** | 21.43 (0.40) | 18.21 (5.19) | 17.26 (8.04) | 16.15 (6.42) | 17.98 (5.39) | 0.194 |
| **Median (Min, Max)** | 21.20 (21.20, 21.90) | 18.50 (2.66, 26.00) | 21.90 (7.98, 21.90) | 18.50 (2.66, 26.00) | 18.50 (2.66, 26.00) |  |
| **Camp size** | | | | | | |
| **Mean (SD)** | 35.33 (16.17) | 50.68 (22.08) | 46.67 (12.70) | 47.63 (22.77) | 50.02 (21.99) | 0.599 |
| **Median (Min, Max)** | 26.00 (26.00, 54.00) | 54.00 (12.00, 77.00) | 54.00 (32.00, 54.00) | 54.00 (12.00, 77.00) | 54.00 (12.00, 77.00) |  |
| **Median camp wealth** | | | | | | |
| **Mean (SD)** | 1.96 (0.24) | 1.92 (0.69) | 1.35 (0.58) | 1.85 (0.85) | 1.90 (0.71) | 0.563 |
| **Median (Min, Max)** | 2.10 (1.68, 2.10) | 1.68 (0.68, 3.55) | 1.68 (0.68, 1.68) | 1.66 (0.68, 3.55) | 1.68 (0.68, 3.55) |  |
| **Household wealth** | | | | | | |
| **Mean (SD)** | 1.75 (0.54) | 2.12 (1.17) | 1.69 (1.06) | 2.00 (1.24) | 2.09 (1.16) | 0.831 |
| **Median (Min, Max)** | 1.90 (1.15, 2.19) | 1.97 (0.18, 5.50) | 1.60 (0.68, 2.80) | 1.76 (0.18, 4.57) | 1.90 (0.18, 5.50) |  |
| **Camp wealth Gini** | | | | | | |
| **Mean (SD)** | 0.16 (0.02) | 0.22 (0.12) | 0.15 (0.03) | 0.22 (0.12) | 0.22 (0.12) | 0.652 |
| **Median (Min, Max)** | 0.17 (0.14, 0.17) | 0.18 (0.00, 0.40) | 0.14 (0.14, 0.19) | 0.17 (0.06, 0.40) | 0.18 (0.00, 0.40) |  |

**DAGs**

We created three base DAGs for the different models (Figures S2-4); from these we checked the implied conditional independencies using the *LAVAAN* package [70]; where there was evidence of a medium-strong effect size (0.1 and above), we added a new path between variables if direct causation could reasonably be inferred. This procedure was repeated until no updates were required; see the SI code for documentation of the full process.

**
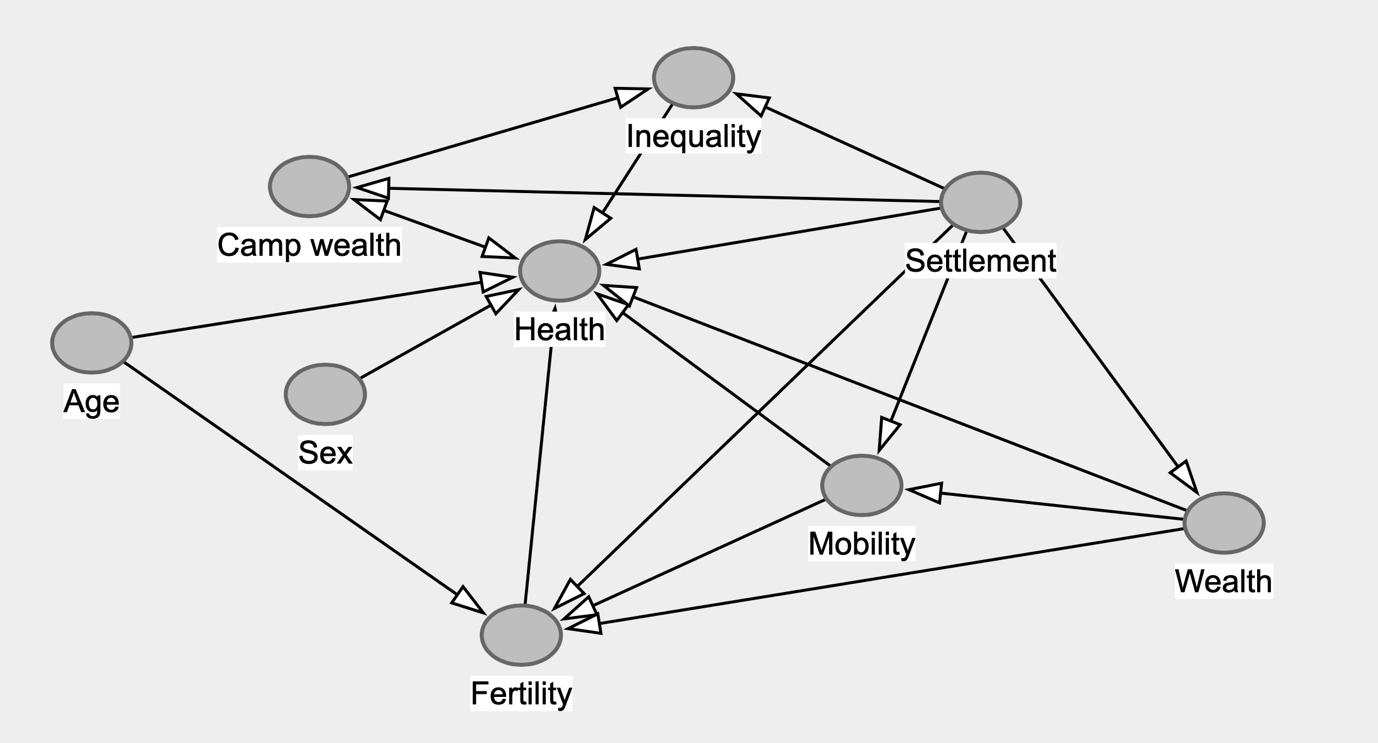
**

**Figure S2: DAG for health outcomes.** Exposure variables were household wealth and wealth Gini, outcome variable is ‘health’ which includes were BMI (body mass index), DBP (diastolic blood pressure), SBP (systolic blood pressure), RBC (red blood cell count), Eosinophils, lymphocytes, neutrophils and NLR (neutrophil – lymphocyte ratio).

**
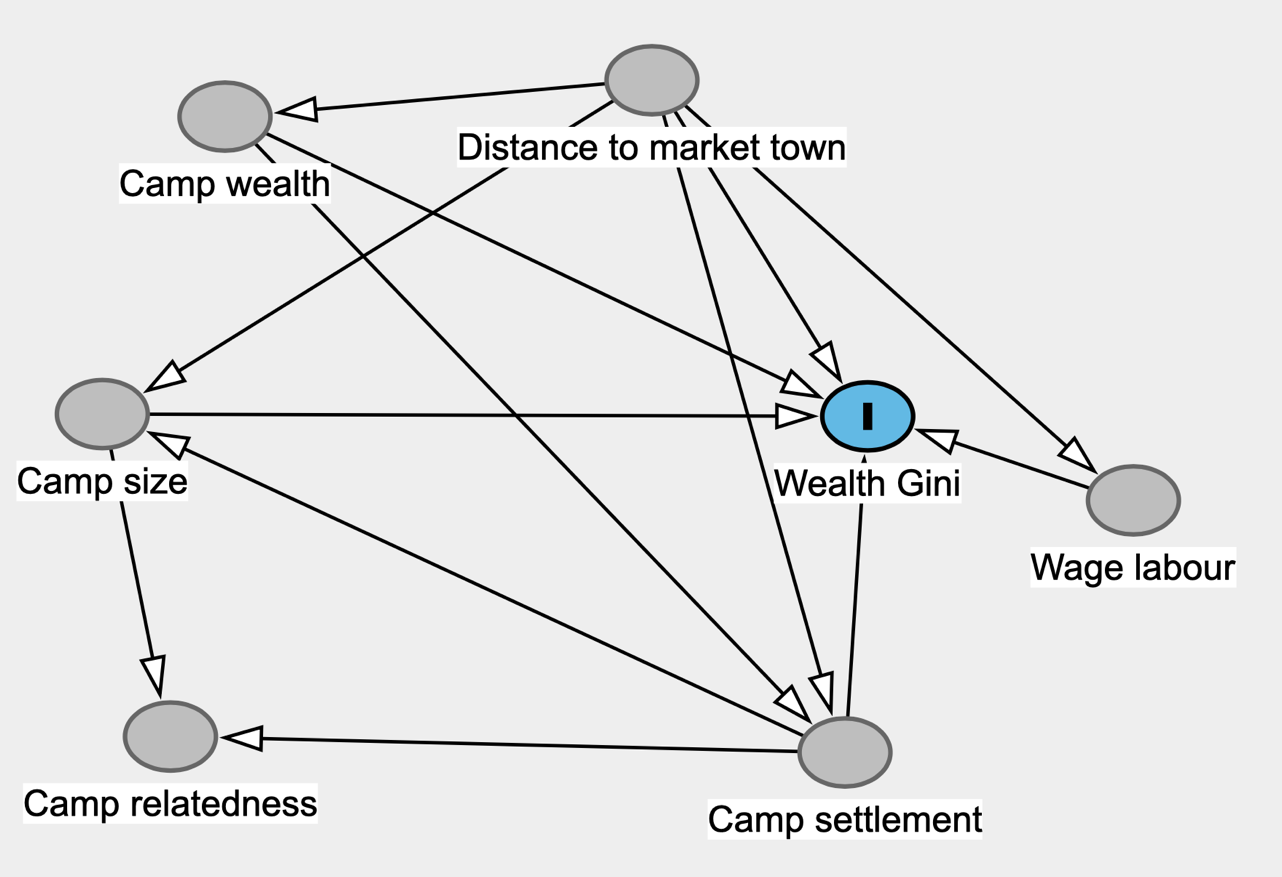
**

**Figure S3:** DAG for camp level exposures (settlement, median camp wealth, camp size, mean camp relatedness, mean proportion of activities spent in wage labour and distance to market town) predicting wealth inequality (wealth Gini, the outcome).


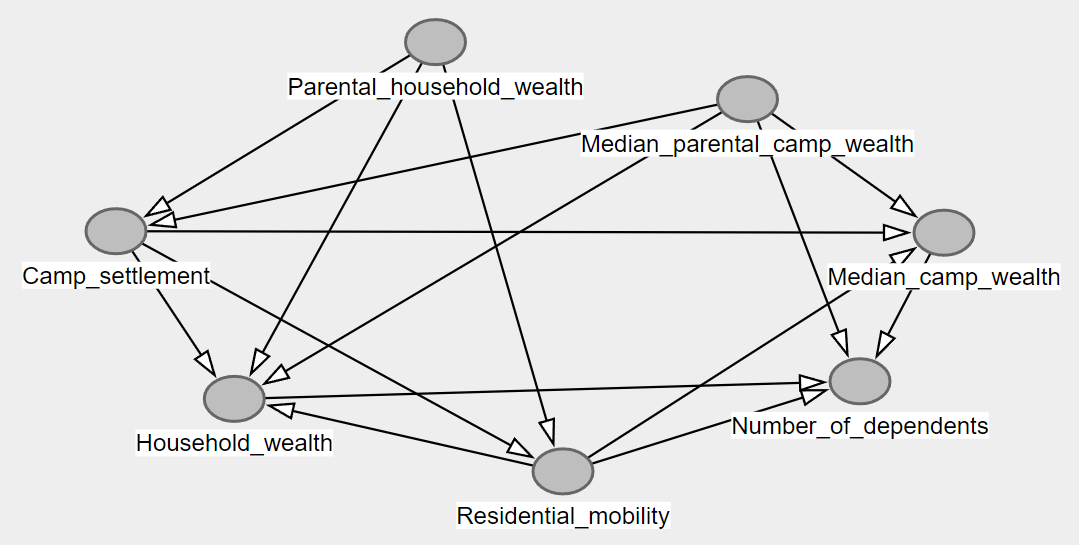


**Figure S4:** DAG for hypothesized causal relationship between parental household wealth (exposure) and adult children’s household wealth (outcome).

**Result tables**

**Adult models**

**Table S4:** BMI by Gini and house wealth

|  | **Estimate** | **SE** | **Z** | **p value** | **95% CI** | |
| --- | --- | --- | --- | --- | --- | --- |
|  |  |  |  |  | **Lower** | **Upper** |
| **(Intercept)** | 17.987 | 0.650 | 27.668 | <0.001 | 16.713 | 19.261 |
| **Mobile Household** | 0.454 | 0.351 | 1.295 | 0.195 | -0.233 | 1.142 |
| **Settled Camp** | 0.519 | 0.345 | 1.502 | 0.133 | -0.158 | 1.196 |
| **Gini Coefficient** | -0.569 | 1.793 | -0.318 | 0.751 | -4.083 | 2.945 |
| **Household wealth** | 0.002 | 0.005 | 0.475 | 0.635 | -0.008 | 0.013 |

**Table S5:** BMI with interaction between Gini and household wealth

|  | **Estimate** | **SE** | **Z** | **p value** | **95% CI** | |
| --- | --- | --- | --- | --- | --- | --- |
|  |  |  |  |  | **Lower** | **Upper** |
| **(Intercept)** | 17.932 | 1.246 | 14.387 | <0.001 | 15.489 | 20.374 |
| **Mobile Household** | 0.455 | 0.351 | 1.296 | 0.195 | -0.233 | 1.143 |
| **Settled Camp** | 0.518 | 0.346 | 1.498 | 0.134 | -0.160 | 1.196 |
| **Gini Coefficient** | -0.378 | 4.062 | -0.093 | 0.926 | -8.340 | 7.583 |
| **Household wealth** | 0.003 | 0.019 | 0.182 | 0.856 | -0.034 | 0.041 |
| **Gini Coefficient:Household wealth** | -0.003 | 0.063 | -0.052 | 0.958 | -0.127 | 0.120 |

**Table S6:** DBP by Gini and house wealth

|  | **Estimate** | **SE** | **Z** | **p value** | **95% CI** | |
| --- | --- | --- | --- | --- | --- | --- |
|  |  |  |  |  | **Lower** | **Upper** |
| **(Intercept)** | 80.057 | 2.826 | 28.332 | >0.001 | 74.519 | 85.595 |
| **Mobile Household** | -0.617 | 1.668 | -0.370 | 0.712 | -3.886 | 2.653 |
| **Settled Camp** | 1.666 | 1.595 | 1.045 | 0.296 | -1.459 | 4.791 |
| **Gini Coefficient** | -10.630 | 8.068 | -1.318 | 0.188 | -26.443 | 5.183 |
| **Household wealth** | 0.023 | 0.023 | 0.981 | 0.327 | -0.023 | 0.069 |

**Table S7:** DBP with interaction between Gini and household wealth

|  | **Estimate** | **SE** | **Z** | **p value** | **95% CI** | |
| --- | --- | --- | --- | --- | --- | --- |
|  |  |  |  |  | **Lower** | **Upper** |
| **(Intercept)** | 70.796 | 5.267 | 13.442 | >0.001 | 60.473 | 81.119 |
| **Mobile Household** | -0.613 | 1.639 | -0.374 | 0.708 | -3.825 | 2.598 |
| **Settled Camp** | 1.411 | 1.571 | 0.898 | 0.369 | -1.669 | 4.490 |
| **Gini Coefficient** | 21.801 | 17.565 | 1.241 | 0.215 | -12.627 | 56.228 |
| **Household wealth** | **0.179** | **0.079** | **2.271** | **0.023** | **0.024** | **0.333** |
| **Gini Coefficient:Household wealth** | **-0.542** | **0.262** | **-2.069** | **0.039** | **-1.055** | **-0.029** |

**Table S8:** SBP by Gini and house wealth

|  | **Estimate** | **SE** | **Z** | **p value** | **95% CI** | |
| --- | --- | --- | --- | --- | --- | --- |
|  |  |  |  |  | **Lower** | **Upper** |
| **(Intercept)** | 104.434 | 3.236 | 32.270 | >0.001 | 98.092 | 110.776 |
| **Mobile Household** | -2.897 | 1.910 | -1.520 | 0.129 | -6.641 | 0.847 |
| **Settled Camp** | 0.380 | 1.826 | 0.210 | 0.835 | -3.199 | 3.958 |
| **Gini Coefficient** | -9.862 | 9.239 | -1.070 | 0.286 | -27.970 | 8.247 |
| **Household wealth** | -0.007 | 0.027 | -0.260 | 0.796 | -0.060 | 0.046 |

**Table S9:** SBP with interaction between Gini and household wealth

|  | **Estimate** | **SE** | **Z** | **p value** | **95% CI** | |
| --- | --- | --- | --- | --- | --- | --- |
|  |  |  |  |  | **Lower** | **Upper** |
| **(Intercept)** | 105.830 | 6.138 | 17.242 | >0.001 | 93.800 | 117.860 |
| **Mobile Household** | -2.898 | 1.910 | -1.517 | 0.129 | -6.640 | 0.845 |
| **Settled Camp** | 0.418 | 1.831 | 0.228 | 0.819 | -3.171 | 4.007 |
| **Gini Coefficient** | -14.749 | 20.470 | -0.720 | 0.471 | -54.869 | 25.372 |
| **Household wealth** | -0.030 | 0.092 | -0.332 | 0.740 | -0.210 | 0.149 |
| **Gini Coefficient:Household wealth** | 0.082 | 0.305 | 0.268 | 0.789 | -0.516 | 0.680 |

**Table S10:** RBC by Gini house wealth - binomial

|  | **Estimate** | **SE** | **Z** | **p value** | **95% CI** | |
| --- | --- | --- | --- | --- | --- | --- |
|  |  |  |  |  | **Lower** | **Upper** |
| **(Intercept)** | -1.877 | 0.889 | -2.112 | 0.035 | 0.027 | 0.874 |
| **Mobile Household** | 0.332 | 0.427 | 0.776 | 0.438 | 0.603 | 3.221 |
| **Settled Camp** | 0.845 | 0.469 | 1.803 | 0.071 | 0.929 | 5.834 |
| **Gini Coefficient** | 4.775 | 2.370 | 2.014 | 0.044 | 1.138 | 12342.350 |
| **Household wealth** | -0.008 | 0.006 | -1.284 | 0.199 | 0.981 | 1.004 |

**Table S11:** RBC by Gini and house wealth - continuous

|  | **Estimate** | **SE** | **Z** | **p value** | **95% CI** | |
| --- | --- | --- | --- | --- | --- | --- |
|  |  |  |  |  | **Lower** | **Upper** |
| **(Intercept)** | 142.437 | 9.011 | 15.807 | >0.001 | 124.776 | 160.098 |
| **Mobile Household** | -3.478 | 4.763 | -0.730 | 0.465 | -12.812 | 5.857 |
| **Settled Camp** | -3.232 | 4.680 | -0.691 | 0.490 | -12.405 | 5.940 |
| **Gini Coefficient** | -58.836 | 24.294 | -2.422 | 0.015 | -106.450 | -11.222 |
| **Household wealth** | 0.003 | 0.070 | 0.037 | 0.970 | -0.135 | 0.141 |

**Table S12:** RBC with continuous interaction between Gini and household wealth

|  | **Estimate** | **SE** | **Z** | **p value** | **95% CI** | |
| --- | --- | --- | --- | --- | --- | --- |
|  |  |  |  |  | **Lower** | **Upper** |
| **(Intercept)** | 130.155 | 17.337 | 7.508 | >0.001 | 96.176 | 164.134 |
| **Mobile Household** | -3.321 | 4.755 | -0.698 | 0.485 | -12.640 | 5.999 |
| **Settled Camp** | -3.670 | 4.699 | -0.781 | 0.435 | -12.878 | 5.539 |
| **Gini Coefficient** | -17.606 | 55.352 | -0.318 | 0.750 | -126.095 | 90.882 |
| **Household wealth** | 0.212 | 0.263 | 0.808 | 0.419 | -0.302 | 0.727 |
| **Gini Coefficient:Household wealth** | -0.703 | 0.848 | -0.828 | 0.407 | -2.365 | 0.960 |

**Table S13:** Neutrophil count by Gini and house wealth - binomial

|  | **Estimate** | **SE** | **Z** | **p value** | **95% CI** | |
| --- | --- | --- | --- | --- | --- | --- |
|  |  |  |  |  | **Lower** | **Upper** |
| **(Intercept)** | -22.580 | 24390.000 | -0.001 | 0.999 | 0.000 | Inf |
| **Mobile Household** | 21.430 | 24390.000 | 0.001 | 0.999 | 0.000 | Inf |
| **Settled Camp** | -0.465 | 1.189 | -0.391 | 0.696 | 0.061 | 6.460 |
| **Gini Coefficient** | -7.249 | 6.178 | -1.173 | 0.241 | 0.000 | 128.924 |
| **Household wealth** | 0.008 | 0.017 | 0.469 | 0.639 | 0.975 | 1.042 |

**Table S14:** Neutrophil count by Gini and house wealth - continuous

|  | **Estimate** | **SE** | **Z** | **p value** | **95% CI** | |
| --- | --- | --- | --- | --- | --- | --- |
|  |  |  |  |  | **Lower** | **Upper** |
| **(Intercept)** | 3.740 | 0.548 | 6.825 | >0.001 | 2.666 | 4.815 |
| **Mobile Household** | 0.700 | 0.309 | 2.265 | 0.024 | 0.094 | 1.306 |
| **Settled Camp** | -0.296 | 0.322 | -0.918 | 0.359 | -0.928 | 0.336 |
| **Gini Coefficient** | -0.642 | 1.566 | -0.410 | 0.682 | -3.712 | 2.428 |
| **Household wealth** | 0.003 | 0.004 | 0.584 | 0.559 | -0.006 | 0.011 |

**Table S15:** Neutrophil count with continuous interaction between Gini and household wealth

|  | **Estimate** | **SE** | **Z** | **p value** | **95% CI** | |
| --- | --- | --- | --- | --- | --- | --- |
|  |  |  |  |  | **Lower** | **Upper** |
| **(Intercept)** | 4.113 | 1.156 | 3.559 | >0.001 | 1.848 | 6.378 |
| **Mobile Household** | 0.695 | 0.309 | 2.247 | 0.025 | 0.089 | 1.301 |
| **Settled Camp** | -0.283 | 0.324 | -0.872 | 0.383 | -0.918 | 0.353 |
| **Gini Coefficient** | -1.963 | 3.930 | -0.499 | 0.617 | -9.665 | 5.740 |
| **Household wealth** | -0.003 | 0.017 | -0.202 | 0.840 | -0.037 | 0.030 |
| **Gini Coefficient:Household wealth** | 0.021 | 0.057 | 0.366 | 0.714 | -0.091 | 0.133 |

**Table S16:** Lymphocyte count by Gini and house wealth - binomial

|  | **Estimate** | **SE** | **Z** | **p value** | **95% CI** | |
| --- | --- | --- | --- | --- | --- | --- |
|  |  |  |  |  | **Lower** | **Upper** |
| **(Intercept)** | -2.177 | 1.395 | -1.561 | 0.118 | 0.007 | 1.744 |
| **Mobile Household** | -0.202 | 0.574 | -0.353 | 0.724 | 0.265 | 2.515 |
| **Settled Camp** | 1.174 | 0.864 | 1.358 | 0.174 | 0.595 | 17.583 |
| **Gini Coefficient** | 2.080 | 3.961 | 0.525 | 0.600 | 0.003 | 18844.644 |
| **Household wealth** | 0.001 | 0.009 | 0.061 | 0.952 | 0.983 | 1.018 |

**Table S17:** Lymphocyte count by Gini and house wealth - continuous

|  | **Estimate** | **SE** | **Z** | **p value** | **95% CI** | |
| --- | --- | --- | --- | --- | --- | --- |
|  |  |  |  |  | **Lower** | **Upper** |
| **(Intercept)** | 2.345 | 0.376 | 6.241 | >0.001 | 1.608 | 3.081 |
| **Mobile Household** | 0.224 | 0.192 | 1.165 | 0.244 | -0.153 | 0.600 |
| **Settled Camp** | 0.205 | 0.211 | 0.972 | 0.331 | -0.208 | 0.619 |
| **Gini Coefficient** | 0.606 | 1.035 | 0.585 | 0.558 | -1.423 | 2.636 |
| **Household wealth** | 0.000 | 0.003 | -0.149 | 0.882 | -0.007 | 0.006 |

**Table S18:** Lymphocyte count with continuous interaction between Gini and household wealth

|  | **Estimate** | **SE** | **Z** | **p value** | **95% CI** | |
| --- | --- | --- | --- | --- | --- | --- |
|  |  |  |  |  | **Lower** | **Upper** |
| **(Intercept)** | 2.972 | 0.715 | 4.156 | 0.000 | 1.570 | 4.374 |
| **Mobile Household** | 0.231 | 0.183 | 1.257 | 0.209 | -0.129 | 0.590 |
| **Settled Camp** | 0.229 | 0.202 | 1.134 | 0.257 | -0.167 | 0.624 |
| **Gini Coefficient** | -1.565 | 2.434 | -0.643 | 0.520 | -6.337 | 3.206 |
| **Household wealth** | -0.010 | 0.011 | -0.993 | 0.321 | -0.031 | 0.010 |
| **Gini Coefficient:Household wealth** | 0.034 | 0.035 | 0.954 | 0.340 | -0.036 | 0.103 |

**Table S19:** Eosinophil count by Gini and house wealth with interaction - binomial

|  | **Estimate** | **SE** | **Z** | **p value** | **95% CI** | |
| --- | --- | --- | --- | --- | --- | --- |
|  |  |  |  |  | **Lower** | **Upper** |
| **(Intercept)** | -0.421 | 2.079 | -0.202 | 0.840 | 0.011 | 38.669 |
| **Mobile Household** | 0.249 | 0.491 | 0.507 | 0.612 | 0.490 | 3.361 |
| **Settled Camp** | 0.148 | 0.518 | 0.286 | 0.775 | 0.420 | 3.202 |
| **Gini Coefficient** | 1.804 | 6.981 | 0.258 | 0.796 | 0.000 | 5320326.000 |
| **Household wealth** | 0.026 | 0.032 | 0.828 | 0.408 | 0.965 | 1.092 |
| **Gini Coefficient:Household wealth** | -0.059 | 0.102 | -0.579 | 0.563 | 0.772 | 1.152 |

**Table S20:** Eosinophil count by Gini and house wealth - continuous

|  | **Estimate** | **SE** | **Z** | **p value** | **95% CI** | |
| --- | --- | --- | --- | --- | --- | --- |
|  |  |  |  |  | **Lower** | **Upper** |
| **(Intercept)** | 0.751 | 0.274 | 2.745 | 0.006 | 0.215 | 1.287 |
| **Mobile Household** | 0.271 | 0.148 | 1.830 | 0.067 | -0.019 | 0.562 |
| **Settled Camp** | 0.103 | 0.161 | 0.640 | 0.522 | -0.212 | 0.418 |
| **Gini Coefficient** | -0.726 | 0.780 | -0.931 | 0.352 | -2.255 | 0.803 |
| **Household wealth** | 0.003 | 0.002 | 1.355 | 0.175 | -0.001 | 0.007 |

**Table S21:** Eosinophil count with continuous interaction between Gini and household wealth

|  | **Estimate** | **SE** | **Z** | **p value** | **95% CI** | |
| --- | --- | --- | --- | --- | --- | --- |
|  |  |  |  |  | **Lower** | **Upper** |
| **(Intercept)** | 0.665 | 0.571 | 1.164 | 0.244 | -0.455 | 1.785 |
| **Mobile Household** | 0.272 | 0.148 | 1.834 | 0.067 | -0.019 | 0.563 |
| **Settled Camp** | 0.100 | 0.162 | 0.618 | 0.537 | -0.217 | 0.416 |
| **Gini Coefficient** | -0.420 | 1.949 | -0.216 | 0.829 | -4.241 | 3.400 |
| **Household wealth** | 0.004 | 0.008 | 0.529 | 0.597 | -0.012 | 0.021 |
| **Gini Coefficient:Household wealth** | -0.005 | 0.028 | -0.171 | 0.864 | -0.060 | 0.051 |

**Table S22:** NLR by Gini and house wealth

|  | **Estimate** | **SE** | **Z** | **p value** | **95% CI** | |
| --- | --- | --- | --- | --- | --- | --- |
|  |  |  |  |  | **Lower** | **Upper** |
| **(Intercept)** | 1.739 | 0.263 | 6.619 | 0.000 | 1.224 | 2.254 |
| **Mobile Household** | 0.075 | 0.149 | 0.504 | 0.614 | -0.217 | 0.367 |
| **Settled Camp** | -0.202 | 0.155 | -1.300 | 0.194 | -0.506 | 0.103 |
| **Gini Coefficient** | -0.632 | 0.751 | -0.842 | 0.400 | -2.104 | 0.840 |
| **Household wealth** | 0.001 | 0.002 | 0.322 | 0.747 | -0.003 | 0.005 |

**Table S23:** NLR with continuous interaction between Gini and household wealth

|  | **Estimate** | **SE** | **Z** | **p value** | **95% CI** | |
| --- | --- | --- | --- | --- | --- | --- |
|  |  |  |  |  | **Lower** | **Upper** |
| **(Intercept)** | 1.382 | 0.552 | 2.505 | 0.012 | 0.301 | 2.463 |
| **Mobile Household** | 0.079 | 0.148 | 0.535 | 0.593 | -0.210 | 0.368 |
| **Settled Camp** | -0.216 | 0.155 | -1.393 | 0.164 | -0.519 | 0.088 |
| **Gini Coefficient** | 0.634 | 1.876 | 0.338 | 0.735 | -3.042 | 4.311 |
| **Household wealth** | 0.006 | 0.008 | 0.795 | 0.427 | -0.009 | 0.022 |
| **Gini Coefficient:Household wealth** | -0.020 | 0.027 | -0.736 | 0.462 | -0.073 | 0.033 |

**Child models**

**Table S24:** BMI by Gini and house wealth

|  | **Estimate** | **SE** | **Z** | **p value** | **95% CI** | |
| --- | --- | --- | --- | --- | --- | --- |
|  |  |  |  |  | **Lower** | **Upper** |
| **(Intercept)** | -1.335 | 0.440 | -3.031 | 0.002 | -2.198 | -0.472 |
| **Settled** | 0.140 | 0.233 | 0.599 | 0.549 | -0.318 | 0.597 |
| **Gini Coefficient** | 0.985 | 1.184 | 0.832 | 0.405 | -1.335 | 3.306 |
| **Household wealth** | 0.003 | 0.004 | 0.799 | 0.424 | -0.004 | 0.010 |

**Table S25:** BMI with continuous interaction between Gini and household wealth

|  | **Estimate** | **SE** | **Z** | **p value** | **95% CI** | |
| --- | --- | --- | --- | --- | --- | --- |
|  |  |  |  |  | **Lower** | **Upper** |
| **(Intercept)** | -1.450 | 0.886 | -1.636 | 0.102 | -3.188 | 0.287 |
| **Settled** | 0.135 | 0.236 | 0.572 | 0.567 | -0.328 | 0.598 |
| **Gini Coefficient** | 1.385 | 2.823 | 0.490 | 0.624 | -4.148 | 6.917 |
| **Household wealth** | 0.005 | 0.014 | 0.352 | 0.724 | -0.023 | 0.033 |
| **Gini Coefficient:Household wealth** | -0.007 | 0.049 | -0.148 | 0.882 | -0.103 | 0.089 |

**Table S26:** RBC by Gini and house wealth - binomial

|  | **Estimate** | **SE** | **Z** | **p value** | **95% CI** | |
| --- | --- | --- | --- | --- | --- | --- |
|  |  |  |  |  | **Lower** | **Upper** |
| **(Intercept)** | -1.729 | 0.963 | -1.795 | 0.073 | 0.027 | 1.173 |
| **Settled** | -1.070 | 0.586 | -1.825 | 0.068 | 0.109 | 1.082 |
| **Gini Coefficient** | 4.715 | 2.842 | 1.659 | 0.097 | 0.425 | 29310.107 |
| **Household wealth** | 0.009 | 0.007 | 1.408 | 0.159 | 0.996 | 1.023 |

**Table S27:** RBC by Gini and house wealth – continuous

|  | **Estimate** | **SE** | **Z** | **p value** | **95% CI** | |
| --- | --- | --- | --- | --- | --- | --- |
|  |  |  |  |  | **Lower** | **Upper** |
| **(Intercept)** | 123.963 | 6.783 | 18.276 | >0.001 | 110.669 | 137.257 |
| **Settled** | 3.517 | 3.598 | 0.977 | 0.328 | -3.536 | 10.569 |
| **Gini Coefficient** | **-38.266** | **18.327** | **-2.088** | **0.037** | **-74.186** | **-2.347** |
| **Household wealth** | -0.053 | 0.058 | -0.914 | 0.361 | -0.168 | 0.061 |

**Table S28:** RBC with continuous interaction between Gini and household wealth

|  | **Estimate** | **SE** | **Z** | **p value** | **95% CI** | |
| --- | --- | --- | --- | --- | --- | --- |
|  |  |  |  |  | **Lower** | **Upper** |
| **(Intercept)** | 114.717 | 13.358 | 8.588 | >0.001 | 88.535 | 140.899 |
| **Settled** | 3.556 | 4.245 | 0.838 | 0.402 | -4.764 | 11.875 |
| **Gini Coefficient** | -7.348 | 44.091 | -0.167 | 0.868 | -93.765 | 79.069 |
| **Household wealth** | 0.082 | 0.207 | 0.395 | 0.693 | -0.323 | 0.486 |
| **Gini Coefficient:Household wealth** | -0.438 | 0.717 | -0.611 | 0.541 | -1.843 | 0.967 |

**Table S29:** Neutrophil count by Gini and house wealth - binomial

|  | **Estimate** | **SE** | **Z** | **p value** | **95% CI** | |
| --- | --- | --- | --- | --- | --- | --- |
|  |  |  |  |  | **Lower** | **Upper** |
| **(Intercept)** | -2.780 | 1.221 | -2.276 | 0.023 | 0.006 | 0.680 |
| **Settled** | -0.314 | 0.607 | -0.517 | 0.605 | 0.222 | 2.401 |
| **Gini Coefficient** | 2.031 | 3.259 | 0.623 | 0.533 | 0.013 | 4531.037 |
| **Household wealth** | 0.000 | 0.010 | -0.042 | 0.967 | 0.980 | 1.020 |

**Table S30:** Neutrophil count by Gini and house wealth - continuous

|  | **Estimate** | **SE** | **Z** | **p value** | **95% CI** | |
| --- | --- | --- | --- | --- | --- | --- |
|  |  |  |  |  | **Lower** | **Upper** |
| **(Intercept)** | 4.725 | 0.686 | 6.887 | 0.000 | 3.380 | 6.070 |
| **Settled** | -0.774 | 0.363 | -2.132 | 0.033 | -1.486 | -0.063 |
| **Gini Coefficient** | 0.669 | 1.846 | 0.363 | 0.717 | -2.948 | 4.287 |
| **Household wealth** | 0.002 | 0.006 | 0.343 | 0.732 | -0.009 | 0.013 |

**Table S31:** Neutrophil count with continuous interaction between Gini and household wealth

|  | **Estimate** | **SE** | **Z** | **p value** | **95% CI** | |
| --- | --- | --- | --- | --- | --- | --- |
|  |  |  |  |  | **Lower** | **Upper** |
| **(Intercept)** | 4.337 | 1.221 | 3.551 | 0.000 | 1.943 | 6.731 |
| **Settled** | -0.787 | 0.366 | -2.152 | 0.031 | -1.504 | -0.070 |
| **Gini Coefficient** | 2.038 | 4.014 | 0.508 | 0.612 | -5.829 | 9.904 |
| **Household wealth** | 0.009 | 0.018 | 0.473 | 0.636 | -0.027 | 0.045 |
| **Gini Coefficient:Household wealth** | -0.024 | 0.063 | -0.384 | 0.701 | -0.148 | 0.099 |

**Table S32:** Eosinophil by Gini and house wealth - binomial

|  | **Estimate** | **SE** | **Z** | **p value** | **95% CI** | |
| --- | --- | --- | --- | --- | --- | --- |
|  |  |  |  |  | **Lower** | **Upper** |
| **(Intercept)** | 0.039 | 0.754 | 0.052 | 0.959 | 0.237 | 4.557 |
| **Settled** | 0.835 | 0.374 | 2.234 | 0.026 | 1.108 | 4.792 |
| **Gini Coefficient** | 0.773 | 2.043 | 0.378 | 0.705 | 0.039 | 118.843 |
| **Household wealth** | 0.005 | 0.007 | 0.764 | 0.445 | 0.992 | 1.018 |

Table S33: Eosinophil by Gini and house wealth - continuous

|  | **Estimate** | **SE** | **Z** | **p value** | **95% CI** | |
| --- | --- | --- | --- | --- | --- | --- |
|  |  |  |  |  | **Lower** | **Upper** |
| **(Intercept)** | 1.199 | 0.412 | 2.909 | 0.004 | 0.391 | 2.006 |
| **Settled** | 0.359 | 0.221 | 1.625 | 0.104 | -0.074 | 0.793 |
| **Gini Coefficient** | 0.173 | 1.114 | 0.155 | 0.877 | -2.011 | 2.356 |
| **Household wealth** | 0.003 | 0.004 | 0.894 | 0.371 | -0.004 | 0.010 |

**Table S34:** Eosinophil with continuous interaction between Gini and household wealth

|  | **Estimate** | **SE** | **Z** | **p value** | **95% CI** | |
| --- | --- | --- | --- | --- | --- | --- |
|  |  |  |  |  | **Lower** | **Upper** |
| **(Intercept)** | 2.592 | 0.706 | 3.671 | 0.000 | 1.208 | 3.975 |
| **Settled** | 0.413 | 0.214 | 1.931 | 0.054 | -0.006 | 0.832 |
| **Gini Coefficient** | **-4.713** | **2.316** | **-2.034** | **0.042** | **-9.253** | **-0.172** |
| **Household wealth** | **-0.021** | **0.011** | **-1.954** | **0.051** | **-0.041** | **0.000** |
| **Gini Coefficient:Household wealth** | **0.085** | **0.036** | **2.367** | **0.018** | **0.015** | **0.156** |

**Table S35:** Lymphocyte count by Gini and house wealth – binomial

|  | **Estimate** | **SE** | **Z** | **p value** | **95% CI** | |
| --- | --- | --- | --- | --- | --- | --- |
|  |  |  |  |  | **Lower** | **Upper** |
| **(Intercept)** | -1.707 | 1.168 | -1.462 | 0.144 | -3.996 | 0.582 |
| **Settled** | 0.714 | 0.618 | 1.155 | 0.248 | -0.498 | 1.925 |
| **Gini Coefficient** | 0.889 | 3.058 | 0.291 | 0.771 | -5.105 | 6.883 |
| **Household wealth** | -0.005 | 0.009 | -0.524 | 0.600 | -0.023 | 0.013 |

**Table S36:** Lymphocyte count by Gini and house wealth - continuous

|  | **Estimate** | **SE** | **Z** | **p value** | **95% CI** | |
| --- | --- | --- | --- | --- | --- | --- |
|  |  |  |  |  | **Lower** | **Upper** |
| **(Intercept)** | 5.267 | 1.226 | 4.295 | >0.001 | 2.864 | 7.670 |
| **Settled** | 0.687 | 0.318 | 2.161 | 0.031 | 0.064 | 1.310 |
| **Gini Coefficient** | -3.007 | 3.849 | -0.781 | 0.435 | -10.550 | 4.536 |
| **Household wealth** | -0.022 | 0.017 | -1.260 | 0.208 | -0.056 | 0.012 |
| **Gini Coefficient:Household wealth** | 0.070 | 0.057 | 1.230 | 0.219 | -0.042 | 0.183 |

**Table S37:** Lymphocyte count with continuous interaction between Gini and household wealth

|  | **Estimate** | **SE** | **Z** | **p value** | **95% CI** | |
| --- | --- | --- | --- | --- | --- | --- |
|  |  |  |  |  | **Lower** | **Upper** |
| **(Intercept)** | 5.267 | 1.226 | 4.295 | >0.001 | 2.864 | 7.670 |
| **Settled** | 0.687 | 0.318 | 2.161 | 0.031 | 0.064 | 1.310 |
| **Gini Coefficient** | -3.007 | 3.849 | -0.781 | 0.435 | -10.550 | 4.536 |
| **Household wealth** | -0.022 | 0.017 | -1.260 | 0.208 | -0.056 | 0.012 |
| **Gini Coefficient:Household wealth** | 0.070 | 0.057 | 1.230 | 0.219 | -0.042 | 0.183 |

**Table S38:** NLR by Gini and house wealth

|  | **Estimate** | **SE** | **Z** | **p value** | **95% CI** | |
| --- | --- | --- | --- | --- | --- | --- |
|  |  |  |  |  | **Lower** | **Upper** |
| **(Intercept)** | 1.441 | 0.237 | 6.087 | >0.001 | 0.977 | 1.905 |
| **Settled** | -0.350 | 0.126 | -2.772 | 0.006 | -0.597 | -0.102 |
| **Gini Coefficient** | -0.420 | 0.642 | -0.654 | 0.513 | -1.679 | 0.839 |
| **Household wealth** | 0.000 | 0.002 | -0.212 | 0.832 | -0.004 | 0.004 |

**Table S39:** NLR with continuous interaction between Gini and household wealth

|  | **Estimate** | **SE** | **Z** | **p value** | **95% CI** | |
| --- | --- | --- | --- | --- | --- | --- |
|  |  |  |  |  | **Lower** | **Upper** |
| **(Intercept)** | 1.300 | 0.423 | 3.070 | 0.002 | 0.470 | 2.129 |
| **Settled** | -0.354 | 0.126 | -2.801 | 0.005 | -0.602 | -0.106 |
| **Gini Coefficient** | 0.078 | 1.391 | 0.056 | 0.956 | -2.648 | 2.803 |
| **Household wealth** | 0.002 | 0.006 | 0.314 | 0.754 | -0.010 | 0.014 |
| **Gini Coefficient:Household wealth** | -0.009 | 0.022 | -0.403 | 0.687 | -0.051 | 0.034 |

**Camp level models**

**Table S40:** Camp Gini Coefficient by Settled Status

|  | **Estimate** | **SE** | **Z** | **p value** | **95% CI** | |
| --- | --- | --- | --- | --- | --- | --- |
|  |  |  |  |  | **Lower** | **Upper** |
| **(Intercept)** | 0.385 | 0.132 | 2.914 | 0.004 | 0.126 | 0.644 |
| **Settled** | 0.164 | 0.086 | 1.910 | 0.056 | -0.004 | 0.331 |
| **Distance to town** | 0.014 | 0.009 | 1.452 | 0.147 | -0.005 | 0.032 |
| **Mean camp wealth** | -0.009 | 0.003 | -2.992 | 0.003 | -0.015 | -0.003 |

**Table S41:** Camp Gini Coefficient by distance to town

|  | **Estimate** | **SE** | **Z** | **p value** | **95% CI** | |
| --- | --- | --- | --- | --- | --- | --- |
|  |  |  |  |  | **Lower** | **Upper** |
| **(Intercept)** | 0.372 | 0.128 | 2.906 | 0.004 | 0.121 | 0.624 |
| **Distance to town** | -0.008 | 0.007 | -1.180 | 0.238 | -0.020 | 0.005 |

**Table S42:** Camp Gini Coefficient by mean wealth

|  | **Estimate** | **SE** | **Z** | **p value** | **95% CI** | |
| --- | --- | --- | --- | --- | --- | --- |
|  |  |  |  |  | **Lower** | **Upper** |
| **(Intercept)** | 0.516 | 0.131 | 3.951 | 0.000 | 0.260 | 0.771 |
| **Mean camp wealth** | -0.005 | 0.002 | -2.017 | 0.044 | -0.010 | 0.000 |
| **Distance to town** | -0.001 | 0.007 | -0.108 | 0.914 | -0.014 | 0.012 |

**Table S43:** Camp Gini Coefficient by % time in wage labour

|  | **Estimate** | **SE** | **Z** | **p value** | **95% CI** | |
| --- | --- | --- | --- | --- | --- | --- |
|  |  |  |  |  | **Lower** | **Upper** |
| **(Intercept)** | 0.136 | 0.108 | 1.263 | 0.207 | -0.075 | 0.348 |
| **Mean wage labour** | 0.540 | 0.149 | 3.637 | 0.000 | 0.249 | 0.832 |
| **Distance to town** | -0.002 | 0.005 | -0.401 | 0.688 | -0.011 | 0.007 |

**Table S44:** Camp Gini Coefficient by camp size

|  | **Estimate** | **SE** | **Z** | **p value** | **95% CI** | |
| --- | --- | --- | --- | --- | --- | --- |
|  |  |  |  |  | **Lower** | **Upper** |
| **(Intercept)** | 0.353 | 0.160 | 2.204 | 0.028 | 0.039 | 0.667 |
| **Camp size** | 0.003 | 0.002 | 1.728 | 0.084 | 0.000 | 0.006 |
| **Settled** | -0.080 | 0.079 | -1.004 | 0.315 | -0.235 | 0.076 |
| **Distance to town** | -0.009 | 0.007 | -1.381 | 0.167 | -0.023 | 0.004 |

**Table S45:** Camp Gini Coefficient by mean relatedness

|  | **Estimate** | **SE** | **Z** | **p value** | **95% CI** | |
| --- | --- | --- | --- | --- | --- | --- |
|  |  |  |  |  | **Lower** | **Upper** |
| **(Intercept)** | 0.307 | 0.155 | 1.975 | 0.048 | 0.002 | 0.611 |
| **Mean camp R** | -0.753 | 0.675 | -1.115 | 0.265 | -2.077 | 0.571 |
| **Camp size** | 0.001 | 0.002 | 0.355 | 0.722 | -0.004 | 0.005 |
| **Settled** | -0.005 | 0.071 | -0.074 | 0.941 | -0.145 | 0.135 |

**Inheritance models**

**Table S46:** Mother's wealth models

|  | **Estimate** | **SE** | **Z** | **p value** | **95% CI** | |
| --- | --- | --- | --- | --- | --- | --- |
|  |  |  |  |  | **Lower** | **Upper** |
| **(Intercept)** | 81.3853 | 14.7136 | 5.531 | 3.18E-08 | 52.547 | 110.223 |
| **Parental wealth** | -0.1147 | 0.182 | -0.63 | 0.529 | -0.471 | 0.242 |

**Table S47:** Father's wealth models

|  | **Estimate** | **SE** | **Z** | **p value** | **95% CI** | |
| --- | --- | --- | --- | --- | --- | --- |
|  |  |  |  |  | **Lower** | **Upper** |
| **(Intercept)** | 51.6976 | 23.9059 | 2.163 | 0.0306 | 4.843 | 98.552 |
| **Parental wealth** | 0.1651 | 0.41 | 0.403 | 0.6872 | -0.638 | 0.9687 |

**Sensitivity analysis**

The following tables repeat the previous models above (Table S4 – S47) exactly expect all analyses now exclude individuals from the camp comprised of one household making the Gini Coefficient 0.

**Adult models**

**Table S48:** BMI by Gini and house wealth

|  | **Estimate** | **SE** | **Z** | **p value** | **95% CI** | |
| --- | --- | --- | --- | --- | --- | --- |
|  |  |  |  |  | **Lower** | **Upper** |
| **(Intercept)** | 18.022 | 0.704 | 25.616 | <2e-16 | 16.643 | 19.400 |
| **Mobile Household** | 0.449 | 0.355 | 1.264 | 0.206 | -0.247 | 1.145 |
| **Settled Camp** | 0.512 | 0.352 | 1.453 | 0.146 | -0.178 | 1.201 |
| **Gini Coefficient** | -0.657 | 1.923 | -0.342 | 0.732 | -4.426 | 3.111 |
| **Household wealth** | 0.003 | 0.005 | 0.473 | 0.636 | -0.008 | 0.013 |

**Table S49:** BMI with interaction between Gini and household wealth

|  | **Estimate** | **SE** | **Z** | **p value** | **95% CI** | |
| --- | --- | --- | --- | --- | --- | --- |
|  |  |  |  |  | **Lower** | **Upper** |
| **(Intercept)** | 17.951 | 1.262 | 14.223 | <2e-16 | 15.478 | 20.425 |
| **Mobile Household** | 0.449 | 0.355 | 1.265 | 0.206 | -0.247 | 1.146 |
| **Settled Camp** | 0.510 | 0.353 | 1.447 | 0.148 | -0.181 | 1.201 |
| **Gini Coefficient** | -0.415 | 4.096 | -0.101 | 0.919 | -8.443 | 7.612 |
| **Household wealth** | 0.004 | 0.019 | 0.195 | 0.846 | -0.034 | 0.041 |
| **Gini Coefficient:Household wealth** | -0.004 | 0.064 | -0.067 | 0.947 | -0.130 | 0.121 |

**Table S50:** DBP by Gini and house wealth

|  | **Estimate** | **SE** | **Z** | **p value** | **95% CI** | |
| --- | --- | --- | --- | --- | --- | --- |
|  |  |  |  |  | **Lower** | **Upper** |
| **(Intercept)** | 79.128 | 3.098 | 25.545 | <2e-16 | 73.057 | 85.199 |
| **Mobile Household** | -0.493 | 1.680 | -0.293 | 0.769 | -3.786 | 2.800 |
| **Settled Camp** | 1.898 | 1.629 | 1.165 | 0.244 | -1.294 | 5.090 |
| **Gini Coefficient** | -8.179 | 8.738 | -0.936 | 0.349 | -25.305 | 8.948 |
| **Household wealth** | 0.023 | 0.024 | 0.960 | 0.337 | -0.024 | 0.069 |

**Table S51:** DBP with interaction between Gini and household wealth

|  | **Estimate** | **SE** | **Z** | **p value** | **95% CI** | |
| --- | --- | --- | --- | --- | --- | --- |
|  |  |  |  |  | **Lower** | **Upper** |
| **(Intercept)** | 70.362 | 5.343 | 13.170 | <2e-16 | 59.891 | 80.833 |
| **Mobile Household** | -0.523 | 1.652 | -0.316 | 0.752 | -3.761 | 2.715 |
| **Settled Camp** | 1.588 | 1.609 | 0.987 | 0.324 | -1.566 | 4.741 |
| **Gini Coefficient** | 22.732 | 17.703 | 1.284 | 0.199 | -11.965 | 57.429 |
| **Household wealth** | **0.174** | **0.079** | **2.195** | **0.028** | **0.019** | **0.330** |
| **Gini Coefficient:Household wealth** | **-0.527** | **0.264** | **-1.997** | **0.046** | **-1.044** | **-0.010** |

**Table S52:** SBP by Gini and house wealth

|  | **Estimate** | **SE** | **Z** | **p value** | **95% CI** | |
| --- | --- | --- | --- | --- | --- | --- |
|  |  |  |  |  | **Lower** | **Upper** |
| **(Intercept)** | 104.158 | 3.558 | 29.272 | <2e-16 | 97.184 | 111.132 |
| **Mobile Household** | -2.860 | 1.930 | -1.482 | 0.138 | -6.643 | 0.923 |
| **Settled Camp** | 0.449 | 1.871 | 0.240 | 0.810 | -3.218 | 4.115 |
| **Gini Coefficient** | -9.133 | 10.038 | -0.910 | 0.363 | -28.807 | 10.541 |
| **Household wealth** | -0.007 | 0.027 | -0.262 | 0.793 | -0.060 | 0.046 |

**Table S53:** SBP with interaction between Gini and household wealth

|  | **Estimate** | **SE** | **Z** | **p value** | **95% CI** | |
| --- | --- | --- | --- | --- | --- | --- |
|  |  |  |  |  | **Lower** | **Upper** |
| **(Intercept)** | 105.628 | 6.240 | 16.929 | <2e-16 | 93.399 | 117.857 |
| **Mobile Household** | -2.855 | 1.930 | -1.480 | 0.139 | -6.637 | 0.926 |
| **Settled Camp** | 0.501 | 1.879 | 0.266 | 0.790 | -3.182 | 4.183 |
| **Gini Coefficient** | -14.315 | 20.675 | -0.692 | 0.489 | -54.838 | 26.208 |
| **Household wealth** | -0.033 | 0.093 | -0.351 | 0.726 | -0.214 | 0.149 |
| **Gini Coefficient:Household wealth** | 0.088 | 0.308 | 0.287 | 0.774 | -0.516 | 0.693 |

**Table S54:** RBC by Gini and house wealth - continuous

|  | **Estimate** | **SE** | **Z** | **p value** | **95% CI** | |
| --- | --- | --- | --- | --- | --- | --- |
|  |  |  |  |  | **Lower** | **Upper** |
| **(Intercept)** | 145.448 | 9.845 | 14.774 | <2e-16 | 126.152 | 164.744 |
| **Mobile Household** | -3.977 | 4.827 | -0.824 | 0.410 | -13.438 | 5.484 |
| **Settled Camp** | -3.811 | 4.760 | -0.801 | 0.423 | -13.141 | 5.518 |
| **Gini Coefficient** | **-66.510** | **26.323** | **-2.527** | **0.012** | **-118.102** | **-14.918** |
| **Household wealth** | 0.002 | 0.071 | 0.035 | 0.972 | -0.136 | 0.141 |

**Table S55:** RBC with continuous interaction between Gini and household wealth

|  | **Estimate** | **SE** | **Z** | **p value** | **95% CI** | |
| --- | --- | --- | --- | --- | --- | --- |
|  |  |  |  |  | **Lower** | **Upper** |
| **(Intercept)** | 132.079 | 17.543 | 7.529 | 0.000 | 97.697 | 166.462 |
| **Mobile Household** | -3.868 | 4.814 | -0.804 | 0.422 | -13.304 | 5.567 |
| **Settled Camp** | -4.380 | 4.786 | -0.915 | 0.360 | -13.760 | 5.001 |
| **Gini Coefficient** | -21.302 | 55.732 | -0.382 | 0.702 | -130.534 | 87.930 |
| **Household wealth** | 0.238 | 0.265 | 0.896 | 0.370 | -0.282 | 0.757 |
| **Gini Coefficient:Household wealth** | -0.788 | 0.857 | -0.920 | 0.358 | -2.468 | 0.892 |

**Table S56:** Neutrophil count by Gini and house wealth - continuous

|  | **Estimate** | **SE** | **Z** | **p value** | **95% CI** | |
| --- | --- | --- | --- | --- | --- | --- |
|  |  |  |  |  | **Lower** | **Upper** |
| **(Intercept)** | 3.854 | 0.604 | 6.379 | 0.000 | 2.670 | 5.038 |
| **Mobile Household** | 0.680 | 0.313 | 2.173 | 0.030 | 0.067 | 1.294 |
| **Settled Camp** | -0.320 | 0.328 | -0.976 | 0.329 | -0.962 | 0.322 |
| **Gini Coefficient** | -0.943 | 1.706 | -0.553 | 0.580 | -4.288 | 2.401 |
| **Household wealth** | 0.003 | 0.004 | 0.597 | 0.550 | -0.006 | 0.011 |

**Table S57:** Neutrophil count with continuous interaction between Gini and household wealth

|  | **Estimate** | **SE** | **Z** | **p value** | **95% CI** | |
| --- | --- | --- | --- | --- | --- | --- |
|  |  |  |  |  | **Lower** | **Upper** |
| **(Intercept)** | 4.191 | 1.174 | 3.570 | 0.000 | 1.890 | 6.492 |
| **Mobile Household** | 0.677 | 0.313 | 2.161 | 0.031 | 0.063 | 1.291 |
| **Settled Camp** | -0.306 | 0.330 | -0.928 | 0.353 | -0.953 | 0.340 |
| **Gini Coefficient** | -2.143 | 3.966 | -0.540 | 0.589 | -9.917 | 5.631 |
| **Household wealth** | -0.003 | 0.017 | -0.168 | 0.866 | -0.037 | 0.031 |
| **Gini Coefficient:Household wealth** | 0.019 | 0.057 | 0.335 | 0.738 | -0.093 | 0.132 |

**Table S58:** Lymphocyte count by Gini and house wealth - continuous

|  | **Estimate** | **SE** | **Z** | **p value** | **95% CI** | |
| --- | --- | --- | --- | --- | --- | --- |
|  |  |  |  |  | **Lower** | **Upper** |
| **(Intercept)** | 2.408 | 0.410 | 5.874 | 0.000 | 1.605 | 3.212 |
| **Mobile Household** | 0.215 | 0.195 | 1.104 | 0.270 | -0.167 | 0.597 |
| **Settled Camp** | 0.191 | 0.216 | 0.887 | 0.375 | -0.231 | 0.614 |
| **Gini Coefficient** | 0.436 | 1.128 | 0.387 | 0.699 | -1.774 | 2.647 |
| **Household wealth** | 0.000 | 0.003 | -0.142 | 0.887 | -0.007 | 0.006 |

**Table S59:** Lymphocyte count with continuous interaction between Gini and household wealth

|  | **Estimate** | **SE** | **Z** | **p value** | **95% CI** | |
| --- | --- | --- | --- | --- | --- | --- |
|  |  |  |  |  | **Lower** | **Upper** |
| **(Intercept)** | 3.010 | 0.729 | 4.128 | 0.000 | 1.581 | 4.439 |
| **Mobile Household** | 0.223 | 0.186 | 1.196 | 0.232 | -0.142 | 0.587 |
| **Settled Camp** | 0.217 | 0.206 | 1.053 | 0.293 | -0.187 | 0.621 |
| **Gini Coefficient** | -1.653 | 2.466 | -0.671 | 0.502 | -6.486 | 3.179 |
| **Household wealth** | -0.010 | 0.011 | -0.961 | 0.337 | -0.031 | 0.011 |
| **Gini Coefficient:Household wealth** | 0.033 | 0.036 | 0.925 | 0.355 | -0.037 | 0.103 |

**Table S60:** Eosinophil count by Gini and house wealth - continuous

|  | **Estimate** | **SE** | **Z** | **p value** | **95% CI** | |
| --- | --- | --- | --- | --- | --- | --- |
|  |  |  |  |  | **Lower** | **Upper** |
| **(Intercept)** | 0.845 | 0.300 | 2.817 | 0.005 | 0.257 | 1.433 |
| **Mobile Household** | 0.258 | 0.150 | 1.722 | 0.085 | -0.036 | 0.551 |
| **Settled Camp** | 0.082 | 0.163 | 0.502 | 0.616 | -0.238 | 0.402 |
| **Gini Coefficient** | -0.980 | 0.846 | -1.158 | 0.247 | -2.639 | 0.679 |
| **Household wealth** | 0.003 | 0.002 | 1.373 | 0.170 | -0.001 | 0.008 |

**Table S61:** Eosinophil count with continuous interaction between Gini and household wealth

|  | **Estimate** | **SE** | **Z** | **p value** | **95% CI** | |
| --- | --- | --- | --- | --- | --- | --- |
|  |  |  |  |  | **Lower** | **Upper** |
| **(Intercept)** | 0.737 | 0.579 | 1.272 | 0.203 | -0.398 | 1.872 |
| **Mobile Household** | 0.259 | 0.150 | 1.727 | 0.084 | -0.035 | 0.552 |
| **Settled Camp** | 0.078 | 0.164 | 0.474 | 0.636 | -0.244 | 0.400 |
| **Gini Coefficient** | -0.592 | 1.964 | -0.301 | 0.763 | -4.440 | 3.257 |
| **Household wealth** | 0.005 | 0.008 | 0.580 | 0.562 | -0.012 | 0.021 |
| **Gini Coefficient:Household wealth** | -0.006 | 0.028 | -0.219 | 0.827 | -0.062 | 0.049 |

**Table S62:** NLR by Gini and house wealth

|  | **Estimate** | **SE** | **Z** | **p value** | **95% CI** | |
| --- | --- | --- | --- | --- | --- | --- |
|  |  |  |  |  | **Lower** | **Upper** |
| **(Intercept)** | 1.743 | 0.290 | 6.011 | 0.000 | 1.175 | 2.311 |
| **Mobile Household** | 0.077 | 0.151 | 0.509 | 0.611 | -0.219 | 0.372 |
| **Settled Camp** | -0.201 | 0.158 | -1.271 | 0.204 | -0.511 | 0.109 |
| **Gini Coefficient** | -0.644 | 0.819 | -0.786 | 0.432 | -2.250 | 0.962 |
| **Household wealth** | 0.001 | 0.002 | 0.318 | 0.750 | -0.004 | 0.005 |

**Table S63:** NLR with continuous interaction between Gini and household wealth

|  | **Estimate** | **SE** | **Z** | **p value** | **95% CI** | |
| --- | --- | --- | --- | --- | --- | --- |
|  |  |  |  |  | **Lower** | **Upper** |
| **(Intercept)** | 1.389 | 0.558 | 2.490 | 0.013 | 0.296 | 2.482 |
| **Mobile Household** | 0.077 | 0.149 | 0.520 | 0.603 | -0.214 | 0.369 |
| **Settled Camp** | -0.218 | 0.157 | -1.388 | 0.165 | -0.525 | 0.090 |
| **Gini Coefficient** | 0.618 | 1.885 | 0.328 | 0.743 | -3.076 | 4.311 |
| **Household wealth** | 0.007 | 0.008 | 0.800 | 0.424 | -0.009 | 0.023 |
| **Gini Coefficient:Household wealth** | -0.020 | 0.027 | -0.740 | 0.459 | -0.074 | 0.033 |

**Child models**

**Table S64:** BMI by Gini and house wealth

|  | **Estimate** | **SE** | **Z** | **p value** | **95% CI** | |
| --- | --- | --- | --- | --- | --- | --- |
|  |  |  |  |  | **Lower** | **Upper** |
| **(Intercept)** | -1.467 | 0.460 | -3.188 | 0.001 | -2.369 | -0.565 |
| **Settled** | 0.180 | 0.237 | 0.760 | 0.447 | -0.284 | 0.645 |
| **Gini Coefficient** | 1.338 | 1.236 | 1.083 | 0.279 | -1.085 | 3.762 |
| **Household wealth** | 0.003 | 0.004 | 0.805 | 0.421 | -0.004 | 0.010 |

**Table S65:** BMI with continuous interaction between Gini and household wealth

|  | **Estimate** | **SE** | **Z** | **p value** | **95% CI** | |
| --- | --- | --- | --- | --- | --- | --- |
|  |  |  |  |  | **Lower** | **Upper** |
| **(Intercept)** | -1.509 | 0.786 | -1.920 | 0.055 | -3.048 | 0.031 |
| **Settled** | 0.178 | 0.239 | 0.744 | 0.457 | -0.291 | 0.647 |
| **Gini Coefficient** | 1.483 | 2.563 | 0.579 | 0.563 | -3.539 | 6.506 |
| **Household wealth** | 0.004 | 0.012 | 0.308 | 0.758 | -0.020 | 0.027 |
| **Gini Coefficient:Household wealth** | -0.003 | 0.041 | -0.064 | 0.949 | -0.083 | 0.077 |

**Table S66:** RBC by Gini and house wealth – continuous

|  | **Estimate** | **SE** | **Z** | **p value** | **95% CI** | |
| --- | --- | --- | --- | --- | --- | --- |
|  |  |  |  |  | **Lower** | **Upper** |
| **(Intercept)** | 125.603 | 7.212 | 17.416 | <2e-16 | 111.468 | 139.738 |
| **Settled** | 3.043 | 3.675 | 0.828 | 0.408 | -4.159 | 10.245 |
| **Gini Coefficient** | **-42.654** | **19.472** | **-2.191** | **0.029** | **-80.818** | **-4.490** |
| **Household wealth** | -0.054 | 0.059 | -0.922 | 0.357 | -0.169 | 0.061 |

**Table S67:** RBC with continuous interaction between Gini and household wealth

|  | **Estimate** | **SE** | **Z** | **p value** | **95% CI** | |
| --- | --- | --- | --- | --- | --- | --- |
|  |  |  |  |  | **Lower** | **Upper** |
| **(Intercept)** | 115.777 | 13.627 | 8.496 | <2e-16 | 89.068 | 142.486 |
| **Settled** | 2.858 | 4.296 | 0.665 | 0.506 | -5.563 | 11.279 |
| **Gini Coefficient** | -9.271 | 44.804 | -0.207 | 0.836 | -97.085 | 78.544 |
| **Household wealth** | 0.100 | 0.210 | 0.474 | 0.635 | -0.312 | 0.511 |
| **Gini Coefficient:Household wealth** | -0.507 | 0.732 | -0.693 | 0.488 | -1.942 | 0.927 |

**Table S68:** Neutrophil count by Gini and house wealth - continuous

|  | **Estimate** | **SE** | **Z** | **p value** | **95% CI** | |
| --- | --- | --- | --- | --- | --- | --- |
|  |  |  |  |  | **Lower** | **Upper** |
| **(Intercept)** | 4.304 | 0.711 | 6.057 | 0.000 | 2.911 | 5.697 |
| **Settled** | -0.648 | 0.363 | -1.784 | 0.074 | -1.359 | 0.064 |
| **Gini Coefficient** | 1.790 | 1.910 | 0.937 | 0.349 | -1.953 | 5.533 |
| **Household wealth** | 0.002 | 0.006 | 0.378 | 0.705 | -0.009 | 0.013 |

**Table S69:** Neutrophil count with continuous interaction between Gini and household wealth

|  | **Estimate** | **SE** | **Z** | **p value** | **95% CI** | |
| --- | --- | --- | --- | --- | --- | --- |
|  |  |  |  |  | **Lower** | **Upper** |
| **(Intercept)** | 4.088 | 1.207 | 3.388 | 0.001 | 1.723 | 6.453 |
| **Settled** | -0.657 | 0.366 | -1.794 | 0.073 | -1.374 | 0.061 |
| **Gini Coefficient** | 2.557 | 3.950 | 0.647 | 0.517 | -5.185 | 10.300 |
| **Household wealth** | 0.006 | 0.018 | 0.330 | 0.741 | -0.029 | 0.041 |
| **Gini Coefficient:Household wealth** | -0.014 | 0.062 | -0.222 | 0.824 | -0.135 | 0.108 |

**Table S70:** Eosinophil by Gini and house wealth - continuous

|  | **Estimate** | **SE** | **Z** | **p value** | **95% CI** | |
| --- | --- | --- | --- | --- | --- | --- |
|  |  |  |  |  | **Lower** | **Upper** |
| **(Intercept)** | 1.218 | 0.440 | 2.767 | 0.006 | 0.355 | 2.081 |
| **Settled** | 0.353 | 0.227 | 1.554 | 0.120 | -0.092 | 0.797 |
| **Gini Coefficient** | 0.120 | 1.190 | 0.101 | 0.920 | -2.212 | 2.451 |
| **Household wealth** | 0.003 | 0.004 | 0.887 | 0.375 | -0.004 | 0.010 |

**Table S71:** Eosinophil with continuous interaction between Gini and household wealth

|  | **Estimate** | **SE** | **Z** | **p value** | **95% CI** | |
| --- | --- | --- | --- | --- | --- | --- |
|  |  |  |  |  | **Lower** | **Upper** |
| **(Intercept)** | 2.585 | 0.716 | 3.612 | 0.000 | 1.182 | 3.988 |
| **Settled** | 0.416 | 0.220 | 1.893 | 0.058 | -0.015 | 0.846 |
| **Gini Coefficient** | **-4.699** | **2.339** | **-2.009** | **0.045** | **-9.283** | **-0.115** |
| **Household wealth** | **-0.021** | **0.011** | **-1.941** | **0.052** | **-0.042** | **0.000** |
| **Gini Coefficient:Household wealth** | **0.086** | **0.037** | **2.349** | **0.019** | **0.014** | **0.157** |

**Table S72:** Lymphocyte count by Gini and house wealth - continuous

|  | **Estimate** | **SE** | **Z** | **p value** | **95% CI** | |
| --- | --- | --- | --- | --- | --- | --- |
|  |  |  |  |  | **Lower** | **Upper** |
| **(Intercept)** | 5.220 | 1.260 | 4.141 | 0.000 | 2.749 | 7.690 |
| **Settled** | 0.696 | 0.328 | 2.119 | 0.034 | 0.052 | 1.340 |
| **Gini Coefficient** | -2.887 | 3.926 | -0.735 | 0.462 | -10.581 | 4.808 |
| **Household wealth** | -0.022 | 0.017 | -1.243 | 0.214 | -0.056 | 0.013 |
| **Gini Coefficient:Household wealth** | 0.070 | 0.058 | 1.216 | 0.224 | -0.043 | 0.183 |

**Table S73:** Lymphocyte count with continuous interaction between Gini and household wealth

|  | **Estimate** | **SE** | **Z** | **p value** | **95% CI** | |
| --- | --- | --- | --- | --- | --- | --- |
|  |  |  |  |  | **Lower** | **Upper** |
| **(Intercept)** | 5.220 | 1.260 | 4.141 | 0.000 | 2.749 | 7.690 |
| **Settled** | 0.696 | 0.328 | 2.119 | 0.034 | 0.052 | 1.340 |
| **Gini Coefficient** | -2.887 | 3.926 | -0.735 | 0.462 | -10.581 | 4.808 |
| **Household wealth** | -0.022 | 0.017 | -1.243 | 0.214 | -0.056 | 0.013 |
| **Gini Coefficient:Household wealth** | 0.070 | 0.058 | 1.216 | 0.224 | -0.043 | 0.183 |

**Table S74:** NLR by Gini and house wealth

|  | **Estimate** | **SE** | **Z** | **p value** | **95% CI** | |
| --- | --- | --- | --- | --- | --- | --- |
|  |  |  |  |  | **Lower** | **Upper** |
| **(Intercept)** | 1.380 | 0.253 | 5.466 | 0.000 | 0.885 | 1.875 |
| **Settled** | -0.332 | 0.129 | -2.568 | 0.010 | -0.585 | -0.078 |
| **Gini Coefficient** | -0.255 | 0.684 | -0.373 | 0.709 | -1.597 | 1.086 |
| **Household wealth** | 0.000 | 0.002 | -0.202 | 0.840 | -0.004 | 0.004 |

**Table S75:** NLR with continuous interaction between Gini and household wealth

|  | **Estimate** | **SE** | **Z** | **p value** | **95% CI** | |
| --- | --- | --- | --- | --- | --- | --- |
|  |  |  |  |  | **Lower** | **Upper** |
| **(Intercept)** | 1.264 | 0.428 | 2.951 | 0.003 | 0.425 | 2.103 |
| **Settled** | -0.336 | 0.130 | -2.592 | 0.010 | -0.590 | -0.082 |
| **Gini Coefficient** | 0.156 | 1.400 | 0.112 | 0.911 | -2.589 | 2.901 |
| **Household wealth** | 0.002 | 0.006 | 0.254 | 0.799 | -0.011 | 0.014 |
| **Gini Coefficient:Household wealth** | -0.007 | 0.022 | -0.337 | 0.736 | -0.050 | 0.035 |

**Camp level models**

**Table S76:** Camp Gini Coefficient by Settled Status

|  | **Estimate** | **SE** | **Z** | **p value** | **95% CI** | |
| --- | --- | --- | --- | --- | --- | --- |
|  |  |  |  |  | **Lower** | **Upper** |
| **(Intercept)** | 0.445 | 0.130 | 3.415 | 0.001 | 0.190 | 0.701 |
| **Settled** | 0.056 | 0.106 | 0.525 | 0.600 | -0.153 | 0.264 |
| **Distance to town** | 0.004 | 0.011 | 0.415 | 0.678 | -0.017 | 0.025 |
| **Mean camp wealth** | -0.006 | 0.004 | -1.579 | 0.114 | -0.013 | 0.001 |

**Table S77:** Camp Gini Coefficient by distance to town

|  | **Estimate** | **SE** | **Z** | **p value** | **95% CI** | |
| --- | --- | --- | --- | --- | --- | --- |
|  |  |  |  |  | **Lower** | **Upper** |
| **(Intercept)** | 0.363 | 0.107 | 3.380 | 0.001 | 0.153 | 0.574 |
| **Distance to town** | -0.006 | 0.006 | -1.104 | 0.270 | -0.017 | 0.005 |

**Table S78:** Camp Gini Coefficient by mean wealth

|  | **Estimate** | **SE** | **Z** | **p value** | **95% CI** | |
| --- | --- | --- | --- | --- | --- | --- |
|  |  |  |  |  | **Lower** | **Upper** |
| **(Intercept)** | 0.484 | 0.109 | 4.452 | 0.000 | 0.271 | 0.697 |
| **Mean camp wealth** | -0.004 | 0.002 | -2.014 | 0.044 | -0.008 | 0.000 |
| **Distance to town** | 0.000 | 0.005 | -0.078 | 0.938 | -0.011 | 0.010 |

**Table S79:** Camp Gini Coefficient by % time in wage labour

|  | **Estimate** | **SE** | **Z** | **p value** | **95% CI** | |
| --- | --- | --- | --- | --- | --- | --- |
|  |  |  |  |  | **Lower** | **Upper** |
| **(Intercept)** | 0.179 | 0.104 | 1.721 | 0.085 | -0.025 | 0.383 |
| **Mean wage labour** | 0.430 | 0.154 | 2.789 | 0.005 | 0.128 | 0.733 |
| **Distance to town** | -0.002 | 0.004 | -0.490 | 0.624 | -0.011 | 0.006 |

**Table S80:** Camp Gini Coefficient by camp size

|  | **Estimate** | **SE** | **Z** | **p value** | **95% CI** | |
| --- | --- | --- | --- | --- | --- | --- |
|  |  |  |  |  | **Lower** | **Upper** |
| **(Intercept)** | 0.444 | 0.132 | 3.373 | 0.001 | 0.186 | 0.703 |
| **Camp size** | 0.002 | 0.001 | 1.514 | 0.130 | -0.001 | 0.004 |
| **Settled** | -0.119 | 0.065 | -1.840 | 0.066 | -0.245 | 0.008 |
| **Distance to town** | -0.011 | 0.005 | -1.923 | 0.055 | -0.021 | 0.000 |

**Table S81:** Camp Gini Coefficient by mean relatedness

|  | **Estimate** | **SE** | **Z** | **p value** | **95% CI** | |
| --- | --- | --- | --- | --- | --- | --- |
|  |  |  |  |  | **Lower** | **Upper** |
| **(Intercept)** | 0.415 | 0.130 | 3.188 | 0.001 | 0.160 | 0.669 |
| **Mean camp R** | -0.928 | 0.542 | -1.713 | 0.087 | -1.991 | 0.134 |
| **Camp size** | 0.000 | 0.002 | -0.228 | 0.820 | -0.004 | 0.003 |
| **Settled** | -0.035 | 0.058 | -0.607 | 0.544 | -0.149 | 0.078 |

**Inheritance models**

**Table S82:** Mother's wealth models

|  | **Estimate** | **SE** | **Z** | **p value** | **95% CI** | |
| --- | --- | --- | --- | --- | --- | --- |
|  |  |  |  |  | **Lower** | **Upper** |
| **(Intercept)** | 81.273 | 15.182 | 5.353 | 0.000 | 51.517 | 111.029 |
| **Parental wealth** | -0.116 | 0.185 | -0.626 | 0.531 | -0.478 | 0.247 |

**Table S83:** Father's wealth models

|  | **Estimate** | **SE** | **Z** | **p value** | **95% CI** | |
| --- | --- | --- | --- | --- | --- | --- |
|  |  |  |  |  | **Lower** | **Upper** |
| **(Intercept)** | 51.698 | 23.906 | 2.163 | 0.031 | 4.843 | 98.552 |
| **Parental wealth** | 0.165 | 0.410 | 0.403 | 0.687 | -0.638 | 0.969 |

**References**

Beck, N., 2008. Diagnostic Hematology. Springer, London.

Bentley, S., Johnson, A., Bishop, C., 1993. A parallel evaluation of four automated hematology analyzers. Am. J. Clin. Pathol. 100, 626–632.

Dyble, M., Salali, G.D., Chaudhary, N., Page, A.E., Smith, D., Thompson, J., Vinicius, L., Mace, R., Migliano, A.B., 2015. Sex equality can explain the unique social structure of hunter-gatherer bands. Science (80-. ). 348, 796–798. https://doi.org/10.1126/science.aaa5139

Greer, P.J., Arber, D.A., Glader, B., List, A.F., Means, R.T., Paraskevas, F., Rodgers, G.M., 2013. Wintrobes Clinical Hemaology, Thirteenth. ed. Lippincott Williams & Wilkins.

Griffin, B.M., 2012. The Cultural Identity of Foragers and the Agta of Palanan, Isabela the Philippines. Anthropos 91, 111–123.

Minter, T., 2010. The Agta of the Northern Sierra Madre: Livelihood strategies and resilience among Philippine hunter-gatherers. Leiden University.

Mitaishvili, R., 2010. The Human Blood: Composition, Typing, Lab Test Interpretation. RM Global Health.

Morris, S.S., Ruel, M.T., Cohen, R.J., Dewey, K.G., de la Brière, B., Hassan, M.N., 1999. Precision, accuracy, and reliability of hemoglobin assessment with use of capillary blood. Am. J. Clin. Nutr. 69, 1243–1248.

Peterson, J.T., 1978. The Ecology of Social Boundaries: Agta Foragers of the Philippines. University of Illinois Press, London.

van Assendelft, O.W., 2002. Calibration, control of hematology analyzers. Adv Adm. Lab 22, 43–47.

Wahed, A., Dasgupta, A., 2015. Hematology and Coagulations: A Comprehensive Review for Board Preparation, Certification and Clinical Practice. Elsevier, London.
